# Unnatural selection of salmon life histories in a modified riverscape

**DOI:** 10.1111/gcb.14896

**Published:** 2019-12-02

**Authors:** Anna M. Sturrock, Stephanie M. Carlson, John D. Wikert, Tim Heyne, Sébastien Nusslé, Joseph E. Merz, Hugh J. W. Sturrock, Rachel C. Johnson

**Affiliations:** ^1^ Center for Watershed Sciences University of California, Davis Davis CA USA; ^2^ Department of Environmental Science, Policy, and Management University of California, Berkeley Berkeley CA USA; ^3^ US Fish and Wildlife Service Lodi CA USA; ^4^ California Department of Fish and Wildlife La Grange CA USA; ^5^ Institute of Marine Sciences University of California Santa Cruz Santa Cruz CA USA; ^6^ Cramer Fish Sciences West Sacramento CA USA; ^7^ Department of Epidemiology & Biostatistics University of California, San Francisco San Francisco CA USA; ^8^ Fisheries Ecology Division Southwest Fisheries Science Center National Marine Fisheries Service Santa Cruz CA USA

**Keywords:** biocomplexity, dam operation, flow alteration, hydrology, juvenile salmon emigration, life‐history diversity, migration phenology, otolith strontium isotopes, portfolio effect

## Abstract

Altered river flows and fragmented habitats often simplify riverine communities and favor non‐native fishes, but their influence on life‐history expression and survival is less clear. Here, we quantified the expression and ultimate success of diverse salmon emigration behaviors in an anthropogenically altered California river system. We analyzed two decades of Chinook salmon monitoring data to explore the influence of regulated flows on juvenile emigration phenology, abundance, and recruitment. We then followed seven cohorts into adulthood using otolith (ear stone) chemical archives to identify patterns in time‐ and size‐selective mortality along the migratory corridor. Suppressed winter flow cues were associated with delayed emigration timing, particularly in warm, dry years, which was also when selection against late migrants was the most extreme. Lower, less variable flows were also associated with reduced juvenile and adult production, highlighting the importance of streamflow for cohort success in these southernmost populations. While most juveniles emigrated from the natal stream as fry or smolts, the survivors were dominated by the rare few that left at intermediate sizes and times, coinciding with managed flows released before extreme summer temperatures. The consistent selection against early (small) and late (large) migrants counters prevailing ecological theory that predicts different traits to be favored under varying environmental conditions. Yet, even with this weakened portfolio, maintaining a broad distribution in migration traits still increased adult production and reduced variance. In years exhibiting large fry pulses, even marginal increases in their survival would have significantly boosted recruitment. However, management actions favoring any single phenotype could have negative evolutionary and demographic consequences, potentially reducing adaptability and population stability. To recover fish populations and support viable fisheries in a warming and increasingly unpredictable climate, coordinating flow and habitat management within and among watersheds will be critical to balance trait optimization versus diversification.

## Graphical abstract

In a heavily dammed Californian river, lower, less variable stream flows were associated with reduced salmon production, and suppressed winter flow cues with reduced early emigration of fry. Otolith records in the returning adults revealed strong selection against early and late migrants, likely resulting from degradation of downstream habitats and increasingly hot summers. Conversely, managed reservoir releases appeared to benefit spring migrants. Yet even with this weakened portfolio, maintaining a broad migratory window increased adult production and reduced variance. This study highlights the importance of freshwater flow and habitat management to recover salmon populations in an increasingly unpredictable climate.

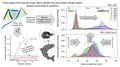

## 1 INTRODUCTION

By responding to seasonal cues in the environment, migratory organisms redistribute themselves across heterogeneous landscapes, spreading risk and diversifying growth opportunities (Scheuerell, Zabel, & Sandford, 2009; Srygley et al., 2010). Within riverine environments, the timing and magnitude of flows influence numerous physical, biological, and ecological processes, including migration phenology for a range of taxa (Sykes, Johnson, & Shrimpton, 2009; Yarnell et al., 2015). However, contemporary populations are often subjected to highly altered flow regimes, with half of the world's large rivers now impounded and regulated (Lehner et al., 2011). With mounting pressure from urbanization and climate change, it is important to quantify and monitor the effects of flow regulation and habitat fragmentation on trait diversity and selection regimes; particularly for at‐risk populations along range margins (Bridle & Vines, 2007).

**Figure 1:**
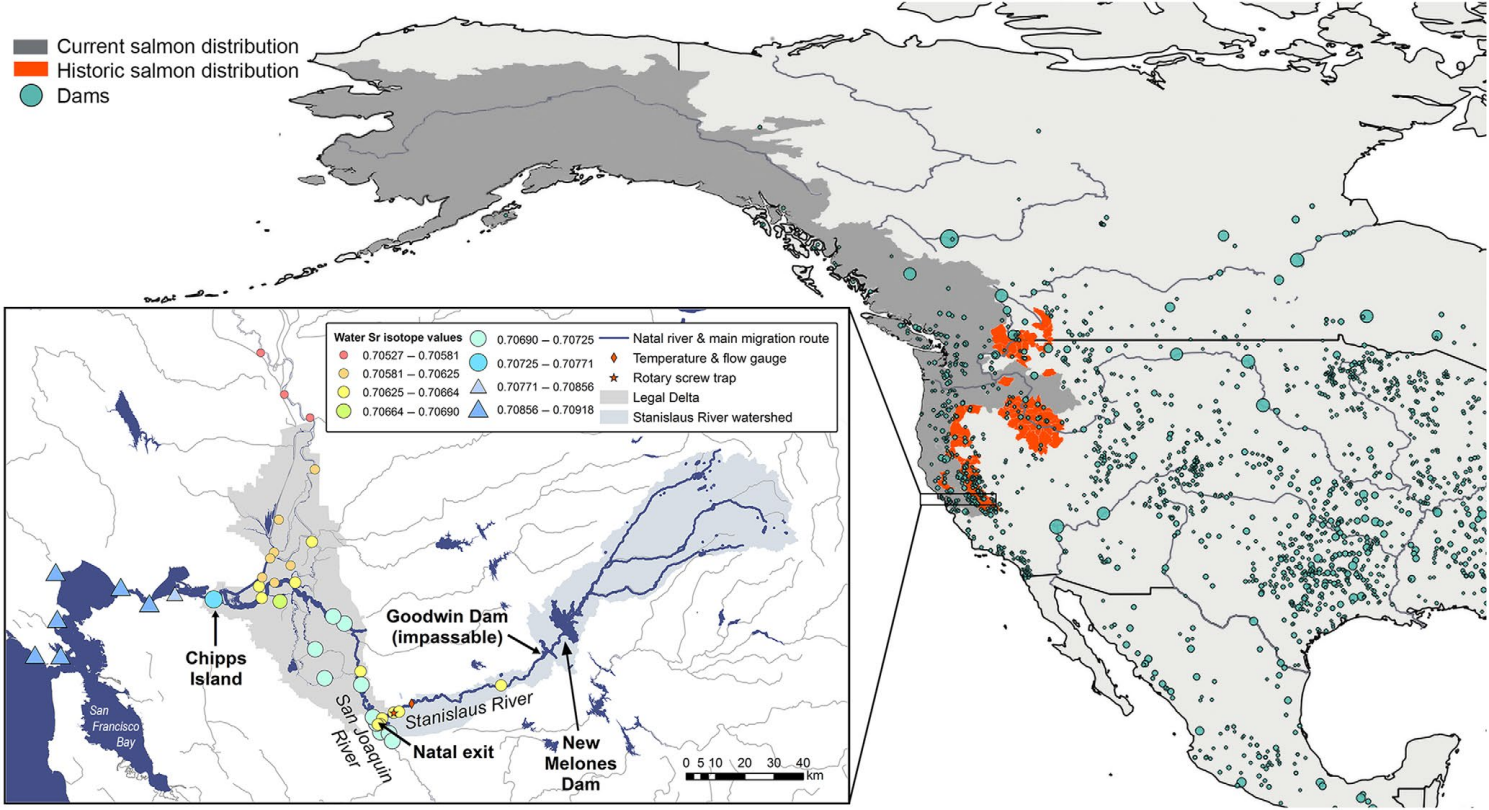
Locations of dams in northwest America (circles, sized by reservoir capacity [typically > 0.1km^3^]. *Source*: GRanD database v1.1, Lehner et al., 2011) relative to current (gray) and historic (red) Chinook salmon distributions (adapted from Behnke, 2002 and Augerot et al., 2005). Inset map of study area: Stanislaus River watershed and the legal boundary of the Sacramento–San Joaquin Delta showing the main emigration pathway from the Stanislaus River to the ocean, the juvenile salmon trapping site, and mean water ^87^Sr/^86^Sr values collected in 1997–2017 (see Supporting Data S1). The isotopic shifts between the Stanislaus River (yellow) and San Joaquin River (turquoise), and between freshwater (0–0.5 ppt, circles) and brackish/marine water (>0.5 ppt, triangles; transition typically located near Chipps Island) were used to reconstruct natal and freshwater exit size in the surviving adults, respectively

Chinook salmon (*Oncorhynchus tshawytscha*) exhibit diverse life histories, having evolved in dynamic and heterogeneous riverscapes (Healey, 1991). Regional disturbances such as wildfires, droughts, and floods (Dettinger, 2011), and variation in ocean upwelling timing and strength (Spence & Hall, 2010) drive adaptive divergence among populations and phenotypic plasticity within populations. The California Central Valley (hereon, “Central Valley”) experiences some of the most extreme climatic variations in North America (Dettinger, 2011; Swain, Langenbrunner, Neelin, & Hall, 2018) and contains the southernmost runs of native Chinook salmon in the world (Figure 1). Salmon in this region exhibit extremely diverse life‐history traits, particularly with respect to adult immigration and juvenile emigration timing (Healey, 1991). Such biocomplexity (Hilborn, Quinn, Schindler, & Rogers, 2003) can translate into differences in population dynamics and emergence of a portfolio effect, whereby variability across the population complex is lower than in its individual components (Schindler et al., 2010). While the stabilizing effect of among‐population diversity has received considerable attention, there is growing appreciation for the importance of within‐population diversity for promoting recovery and resilience at watershed scales (Greene, Hall, Guilbault, & Quinn, 2010; Johnson, Grorud‐Colvert, Sponaugle, & Semmens, 2014).

After the Colorado River, the Sacramento–San Joaquin River system is considered the most regulated and fragmented in North America (Dynesius & Nilsson, 1994), and Central Valley salmon now exhibit a weakened portfolio effect signaled by increasing among‐population synchrony and interannual variability in adult returns (Carlson & Satterthwaite, 2011; Griffiths et al., 2014; Satterthwaite & Carlson, 2015). The region experiences a Mediterranean climate with natural flows driven by winter storm events, late spring snow melt, and a hot, dry summer (SJRGA, 2011; Yarnell et al., 2015). Historically, Central Valley salmon accessed high elevation and spring‐fed streams to escape the warm valley floor, but most of these habitats are now blocked by large, impassable dams (Yoshiyama, Gerstung, Fisher, & Moyle, 2001; Figure 1). Moreover, the timing and magnitude of flows are often highly altered, and largely driven by managed reservoir releases (Zimmerman et al., 2018). In the semi‐arid, southernmost reaches of the Central Valley, flood protection, irrigated agriculture, and urbanization result in water demands often exceeding natural supply (Zimmerman et al., 2018), earning the San Joaquin River the title of “America's Most Endangered River” in 2014 (American Rivers, 2014).

While preserving and restoring life‐history diversity remains an integral goal of many conservation programs, empirical methods to identify controls on and changes in trait expression are scarce. Furthermore, relating early life‐history diversity to adult recruitment requires tracking individuals across life stages, which is particularly challenging for migratory species. Most juvenile salmon survival estimates are generated through physical tagging of larger bodied individuals (often hatchery smolts >80 mm fork length [FL], e.g., Buchanan, Brandes, & Skalski, 2018), yet most juveniles leave the natal tributary as fry (typically 30–55 mm FL), which exhibit markedly different rearing behaviors and sea readiness (Williams, 2006). The chemistry of fish otoliths (ear stones) can act as a natural tag, allowing reconstruction of natal origin and movements among freshwater habitats (Hamann & Kennedy, 2012). In the Central Valley, strontium isotope ratios (^87^Sr/^86^Sr) are a powerful natural tag because they remain relatively stable over time, vary extensively among habitats, and are faithfully recorded in fish otoliths (Barnett‐Johnson, Pearson, Ramos, Grimes, & MacFarlane, 2008). As juvenile salmon otoliths grow incrementally and proportionally to body size, they also provide a useful tool to reconstruct age, size, and growth trajectories in surviving individuals to reveal differences in the success of particular phenotypes among years and populations (Phillis, Sturrock, Johnson, & Weber, 2018; Sturrock et al., 2015; Woodson et al., 2013).

Here, we compiled streamflow records, juvenile and adult salmon abundance data, and otolith ^87^Sr/^86^Sr records in surviving adults to explore how flow alteration influences juvenile salmon emigration behavior, life‐history diversity, and survival. We focus on the Stanislaus River in the San Joaquin River Basin, a highly regulated, snow‐fed river at the southernmost extent of the native species range, where >50% of historical salmon habitats are now blocked by dams (Yoshiyama et al., 2001; Figure 1). Our primary research questions were: (a) Do altered natal stream flows influence juvenile salmon emigration timing (phenotype expression)? (b) Do flows experienced during juvenile rearing influence productivity? (c) Do survival probabilities differ among migratory phenotypes and/or among years? Finally, we discuss the study implications in a changing climate, and explore the extent to which management actions that alter phenotype survival rates might influence population size and stability.

## 2 MATERIALS AND METHODS

### 2.1 Influence of altered streamflow on juvenile salmon phenotype expression

#### 2.1.1 Environmental data

Mean daily flows and maximum daily temperatures were measured in the lower Stanislaus River at Ripon (Figure 1, USGS gauge 11303000, www.waterdata.usgs.gov/nwis). We used the Indicators of Hydrologic Alteration software (Richter, Baumgartner, Powell, & Braun, 1996) to compare daily mean flow and monthly median flow before versus after construction of the largest dam in the watershed (New Melones, which impounds 3 km^3^ or >200% of average annual runoff, Figure 1). We also compared mean daily flow at Ripon with unimpaired daily flow (an estimate of natural runoff that would have been measured at Goodwin Dam in the absence of anthropogenic alteration; CDEC Station “GDW,” http://cdec.water.ca.gov; Figure 1).

#### 2.1.2 Emigrant sampling

Juvenile salmon were sampled by rotary screw traps as they emigrated from the Stanislaus River in 1996–2014 (Figure 1), providing estimates of emigrant size, phenology, and abundance. We excluded 1997 and 2006 because high winter flows precluded sampling for extended periods of time. Migratory phenotypes were classified using FL at natal exit: fry (≤55 mm), parr (>55 to 75 mm), smolts (>75 mm), and yearlings (>75 mm in January, >100 mm in February, and >120 mm in March–June). While these size classifications are somewhat arbitrary and there was rarely a distinct pulse of “parr,” we retained this phenotype to be consistent with other studies (e.g., Miller, Gray, & Merz, 2010), and to characterize the transitional period between fry and smolt emigration (Figure S1). Yearlings were excluded from further analysis as they were rarely observed (<0.05% of emigrants), but warrant future study given potentially disproportionate survival rates and influence on ocean arrival timings.

Marked releases were used to develop a statistical model of trap efficiency based on fish size, flow, and emigration year. Daily and annual passage estimates (±95% CI) were generated using simulated catch and trap efficiencies, incorporating both sampling (catch) and estimation (efficiency model) error (detailed in Sturrock et al., 2015; Zeug, Sellheim, Watry, Wikert, & Merz, 2014). Periods of peak emigration were described using the interquartile range and median passage date.

#### 2.1.3 Phenotype expression

To explore environmental and demographic drivers of juvenile phenotype expression, we modeled the fraction of the population that emigrated as fry, parr or smolts each year using cumulative link models (R package “VGAM” [Yee, 2010]), akin to an ordered multinomial logistic regression assuming nonindependence among groups (McCullagh, 1980). We ran the models assuming proportional and nonproportional odds (with and without parallelism enforced, respectively). As the ordinal outcome has three levels, two cumulative logits were modeled: fry versus parr or smolt, and fry or parr versus smolt.

Model covariates included flow and temperature conditions across the 6 month rearing and emigration period (standard deviation [*SD*], coefficient of variation [CV], and mean of daily records between January 1 and June 30 each year). To capture both flow volume and variability in a single metric, we calculated “within‐season flow variability” as the January–June weekly moving range (specifically the average of a daily moving 7 day window of max[flow] − min[flow]). We also included the number of spawners from the previous fall (Grandtab estimates from www.calfish.org) as a proxy for potential juvenile production and instream competition. Correlated terms (*r* > .6 or < −.6) were analyzed in separate models, then we selected the highest performing model based on likelihood ratio tests and pseudo‐*r* ^2^ values.

### 2.2 Influence of natal streamflow on cohort strength

To explore influences of streamflow on cohort strength, we correlated mean January–June flows and within‐season flow variability against the annual number of juvenile emigrants and adult recruits produced by each cohort. Recruitment was estimated as escapement minus strays, corrected for return age and harvest (see Supporting Methods and Table S2). Straying rates were estimated using otolith natal assignments where available and Constant Fractional Marking (CFM) tag recovery data for other years (e.g., Kormos, Palmer‐Zwahlen, & Low, 2012). For years without any straying estimates, we extrapolated the first and last available CFM estimates (see Supporting Information). For years with both CFM and otolith straying estimates, recruitment estimates were similar using either data source (mean difference < 2%, Figure S3).

### 2.3 Differences in survival between juvenile migratory phenotypes

To reconstruct natal origin and the early life histories of surviving adults born in the Stanislaus River, sagittal otoliths were extracted from 785 postspawned Chinook salmon carcasses in 2001–2013 (Table S2). Adults were aged using scale annuli to cohort match them with the juvenile sample and the flow conditions they had experienced as juveniles. Otolith ^87^Sr/^86^Sr was measured across the juvenile portion of the adult otolith along a standardized 90° transect (Woodson et al., 2013) using laser ablation multiple collector inductively coupled plasma mass spectrometry (UC Davis Interdisciplinary Center for Plasma Mass Spectrometry). Adult natal origin was assigned using the otolith ^87^Sr/^86^Sr values measured immediately after emergence in a linear discriminant function analysis (Barnett‐Johnson et al., 2008; Sturrock et al., 2015). We primarily analyzed unmarked fish, but a subset of tagged hatchery fish were included blind to assess assignment accuracy (*n* = 36; 100% correctly classified as strays; 97% to the correct hatchery‐of‐origin). Strays from other rivers or hatcheries (51% of the 749 unmarked adults) were excluded to ensure that all adults included in the analysis had been born in the Stanislaus River and were comparable with the juvenile trapping data. In the otoliths of returning adults, deviations from natal and freshwater isotopic ranges (obtained from water samples and otoliths from known‐origin juveniles, see Supporting Data S1) were used to identify major habitat transitions (specifically, natal and freshwater exit, Figure 1; see Supporting Information).

To reconstruct juvenile FLs in the surviving adults, we built a broken‐stick model (R package “segmented”) using FL and otolith radius data from juveniles within the same evolutionarily significant unit (*n* = 294, Figure S2). We applied a biological intercept of 30 mm so that size at first feeding (~32 mm) reflected observations in the literature (Titus, Volkoff, & Snider, 2004). We used the model to predict FL at natal and freshwater exits in the returning adults, and resampled the residuals to simulate a distribution of potential FLs for each cohort (*n* = 2,000) and generate 95% confidence intervals (CI) around phenotype contributions and abundances in the adult survivors.

Adult recruitment estimates were separated into migratory phenotypes using the proportions of the returning adults that had emigrated from the natal stream as fry, parr, and smolts based on otolith reconstructions. We generated 95% CI using the error in the FL back‐calculation model (Figure S2). Survival probabilities were estimated by dividing the number of recruits by the number of juveniles per size class and year, resampling the simulated data to generate 95% CI that incorporated uncertainty in both juvenile abundance estimates and FL reconstructions. If simulated survival probabilities exceeded 100%, they were assumed spurious and capped at 100%. This was primarily an issue in 2008 when low catches of parr (*n* = 4 individuals) resulted in greater uncertainty in passage expansions. Note that our survival probabilities should not be considered instantaneous mortality rates, as they were not normalized to the time between natal exit and adult recruitment (typically longer for fry, shorter for smolts).

Survival probabilities were compared among phenotypes using Welch’s ANOVA and Games‐Howell post hoc tests, and by randomly resampling simulated survival estimates (the fraction of draws [*n* = 100,000] exhibiting a survival difference greater than zero representing a one‐tailed *p* value; see Supporting Methods).

## 3 RESULTS

### 3.1 Altered river flows shaped trait expression and suppressed fry dispersal

**Figure 2:**
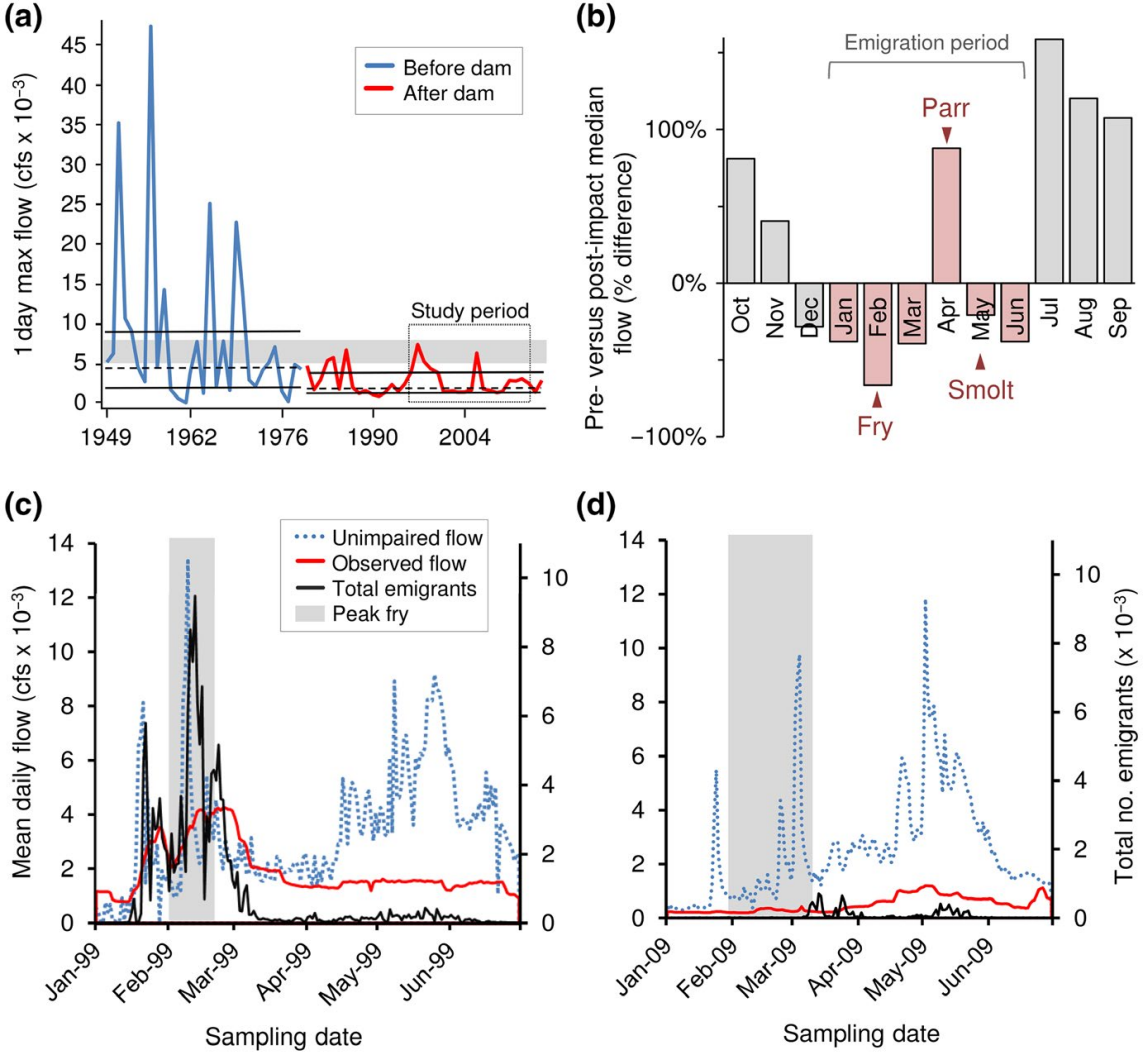
Altered flows influence juvenile salmon emigration phenology. (a) Annual peak daily flows before (blue) versus after (red) major dam construction, indicating median (dashed line), interquartile range (solid lines), minimum “channel maintenance” flows (suggested range of flows needed to maintain spawning grounds on the Stanislaus River; gray box [Kondolf et al., 2001]). (b) Percent difference in median monthly flows following dam construction (peak emigration timing of fry, parr, and smolts indicated by arrows, Table S3). (c,d) Observed (red) and unimpaired (blue, dashed) daily flows, relative to total daily juvenile passage (black) in wet (1999) and dry (2009) years that represented maximum and minimum fry expression, respectively (Figure 3). Period of peak fry emigration (IQR) indicated by shaded area

The Stanislaus River has been profoundly altered by >40 dams with a collective capacity to store ca. 240% of the average annual runoff (Kondolf, Falzone, & Schneider, 2001). Anthropogenic alterations began in the mid‐1800s, preceding flow records and prohibiting true “pre‐impact” comparisons. However, comparing ca. 30 year periods before and after major dam construction (1949–1979 vs. 1980–2016), postimpact flows were 65% less variable (CV of daily flows = 1.78 vs. 1.13 and mean within‐season flow variability = 775 vs. 272 cfs) and annual peak flows were significantly reduced (Welch's test *F*_1,40_ = 4.3, *p* = .046 after adjustment for temporal autocorrelation, Figure 2a). Flow reductions were the most extreme in winter, coinciding with peak fry emigration (Figure 2b).

Fall‐run Chinook salmon primarily leave freshwater as subyearlings, their emigration portfolio dominated by newly emerged fry that disperse from the natal stream during turbid winter storm events, and a smaller pulse of smolts that emigrate with spring recession flows following peak snow melt (Williams, 2006; Yarnell et al., 2015). On the Stanislaus River, these cues are now largely artificial and often decoupled from the unimpaired hydrograph (Figure 2c,d; Figure S4), with fry emigrating during winter flood releases (January–March, when present, Figure 2c,d), and parr and smolts with managed flows in April–May designed to improve their downstream survival rates (SJRGA, 2011; Figure 2b; Table S3).

Emigration rates tended to increase during periods of flow change (Figure 2c,d; Figure S5). The model results suggest that phenotype expression is primarily driven by flow volume and variability, mediated by density‐dependent processes (Table 1). Specifically, greater within‐season flow variability and spawner densities were associated with earlier emigration and a larger fraction of the population leaving as fry. The model explained 66% of the variation in phenotype composition, predicting fry and smolt expression more successfully than parr (*r* ^2^ ≥ .74, vs. .42, respectively, Figure S6).

**Table 1:** Model outputs predicting juvenile salmon phenotype expression (annual proportion of the populating emigrating from the natal stream as fry, parr, or smolts). See footnote (a) for predictive equations by phenotype. Note that we also ran the model using alternative flow and temperature metrics (see above), but they produced significantly lower fit

	Variable	Coefficient	*SE*	*z* value	*p *value
logit (>fry)	Intercept (*a*)	22.03	1.15	19.20	<.0001
	Within‐season flow variability (*f*)	−3.00	0.17	−17.56	<.0001
	Spawner density (*s*)	−0.70	0.06	−11.25	<.0001
logit (≥smolt)	Intercept (*a'*)	19.13	1.14	16.84	<.0001
	Within‐season flow variability (*f'*)	−2.22	0.17	−12.89	<.0001
	Spawner density (*s'*)	−0.97	0.06	−15.98	<.0001

^a^% fry = $1-\left(\frac{e^{\left(a+Xf+Ys\right)}}{1+e^{\left(a+Xf+Ys\right)}}\right)$; % smolt = $\frac{e^{\left(a^{\prime }+Xf^{\prime }+Ys^{\prime }\right)}}{1+e^{\left(a^{\prime }+Xf^{\prime }+Ys^{\prime }\right)}}$; % parr = 1 − % fry − % smolt, where *X* represents log‐transformed within‐season flow variability (mean January–June weekly moving range) and *Y* represents log‐transformed number of spawners. Coefficients (lowercase letters) defined in Table 1.

**Figure 3:**
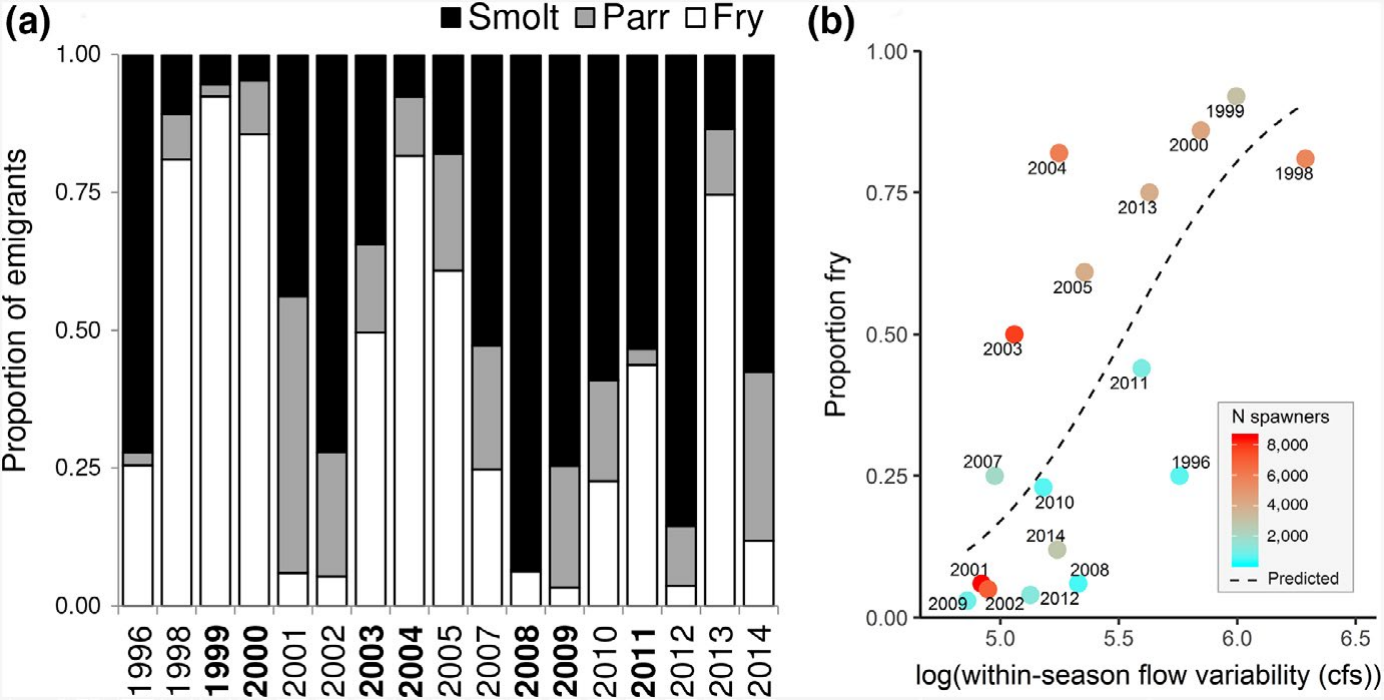
Interannual patterns in juvenile salmon phenotype expression. (a) The proportion of fry (white), parr (gray), and smolt (black) emigrants (cohorts paired with adult otolith reconstructions highlighted in bold; 1997 and 2006 excluded because high flows precluded sampling for much of the winter). (b) Relationship between within‐season flow variability and proportion of fry migrants, colored by the number of parental spawners (proxy for crowding); predicted line based on model output (Table 1), assuming mean spawner density for all years

During the juvenile monitoring period (1996–2014), observed daily flows were typically lower and less variable than unimpaired flows, and winter pulse flows often reduced or absent (Figure 2d). In years lacking winter pulse flows, salmon tended to emigrate later, larger, and in lower numbers (Figure 2d). Overall, fry represented <50% of the emigrants in >50% of the years examined (Figure 3a), contrasting with the fry‐dominated portfolios typical for the region (Brandes & McLain, 2001; Williams, 2006). While crowding and instream carrying capacity (inferred by spawner density) played an important role in emigration timing, flows ultimately determined whether large numbers of fry were triggered and/or displaced downstream or not, with years of high spawner densities and low flows (e.g., 2001–2002) associated with low numbers of fry migrants (Figure 3b). Based on spawner densities and within‐season flow variability (recognizing that a host of other parameters will have also changed), predicted fry expression was 62% lower following major dam construction (median = 95% for 1953–1979 vs. 36% for 1980–2016; equation in Table 1).

### 3.2 Lower, less variable flows were associated with reduced fish production

**Figure 4:**
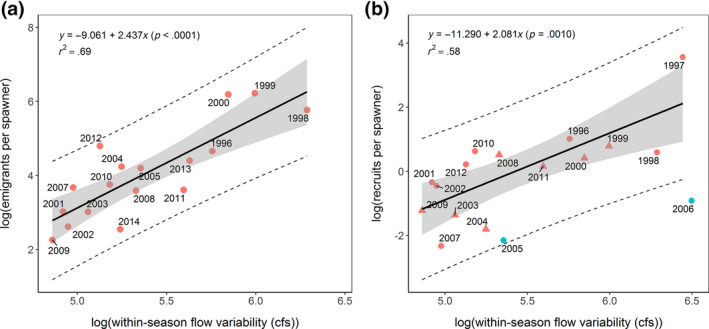
Relationship between salmon abundance and natal streamflow, indicated by within‐season flow variability (mean January–June weekly moving range) versus (a) emigrants‐per‐spawner, and (b) recruits‐per‐spawner, labeled by emigration year. 95% CI indicated by shaded area (fit) and dashed lines (prediction). Cohorts that experienced unusually poor ocean conditions (Lindley et al., 2009; turquoise symbols) were excluded from the regression lines shown in (b). Provenance of spawners (used to remove strays and estimate the number of adult recruits) were based on otolith ^87^Sr/^86^Sr ratios (triangles) or tag recoveries (circles)

**Figure 5:**
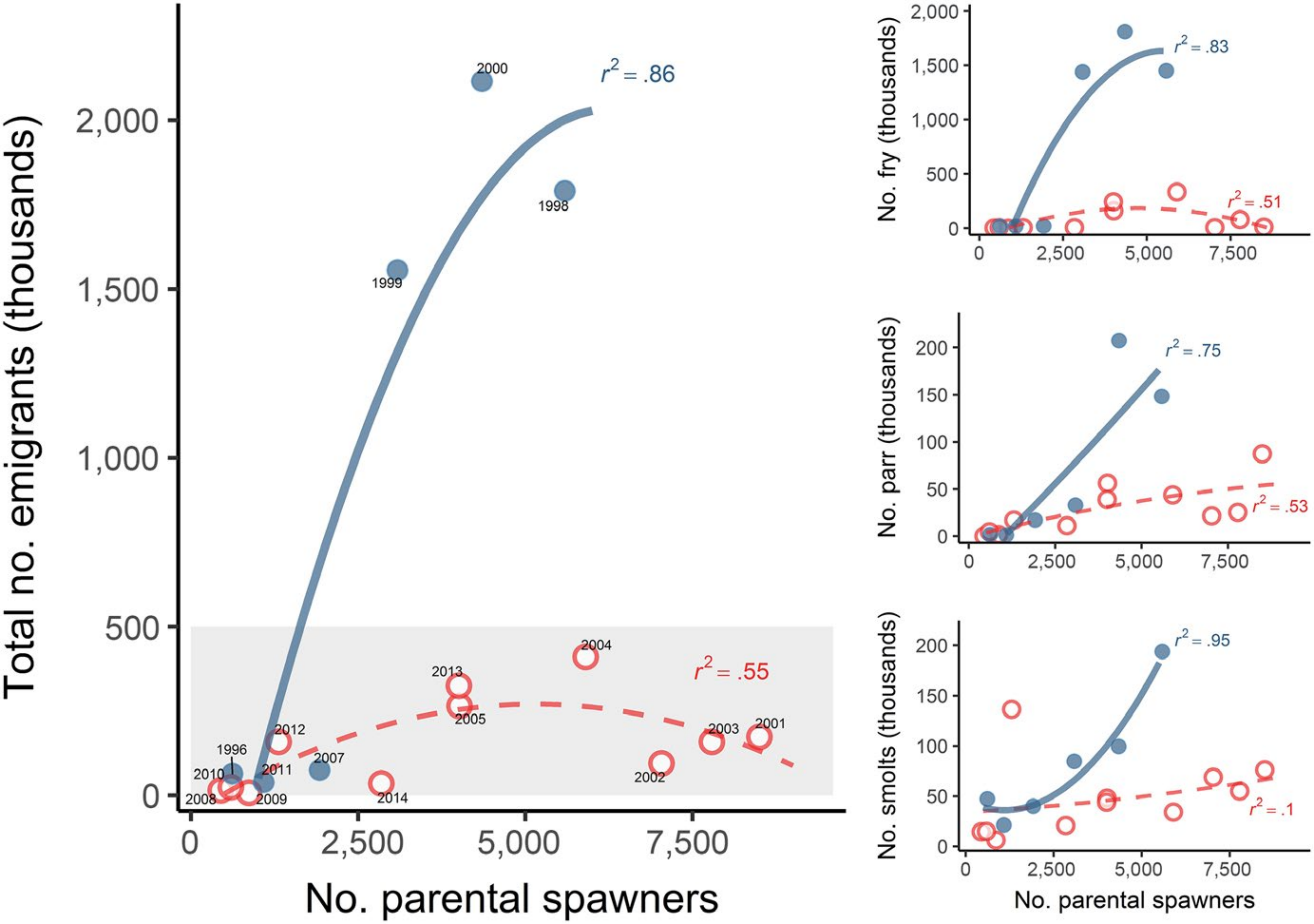
Emigrants‐per‐spawner were typically more abundant in wetter years (blue circles, solid lines; defined as mean January–June natal stream flows ≥987 cfs) than drier years (red open circles, dashed lines), both overall (main plot) and separated into life stages (inset plots), suggesting a flow‐mediated carrying capacity in the natal stream (dry year capacity suggested by shaded box). Predicted lines are from second‐order polynomial regression models, fitted in R

Lower, less variable flows were associated with fewer juvenile emigrants and fewer adult recruits (Figures 4 and 5). Drier years (coarsely defined as mean January–June flows <987 cfs, based on average flows across sampling seasons) produced significantly fewer emigrants‐per‐spawner (means = 45 vs. 249; *F*_1,15_ = 9.30, *p* = .008). Annual abundances of emigrants‐per‐spawner were positively related to within‐season flow variability (*r* ^2^ = .69, *p* < .0001, Figure 4) and mean rearing flows (*r* ^2^ = .58, *p* < .001). As fry often disperse in such high numbers, total emigrant abundances are often driven by fry expression rates. Yet parr and smolt abundances were also typically higher in wet years, suggesting a flow‐mediated carrying capacity for juvenile Chinook salmon in the natal stream (Figure 5). Recruits‐per‐spawner was also positively related to within‐season flow variability during the juvenile rearing period (*r* ^2^ = .58, *p* = .0010 excluding the 2005–2006 cohorts that entered the ocean during exceptionally poor ocean conditions [Lindley et al., 2009]; *r* ^2^ = .33, *p* = .016 with all years included, Figure 4b).

### 3.3 Selection against the tails homogenized the life‐history portfolio

**Figure 6:**
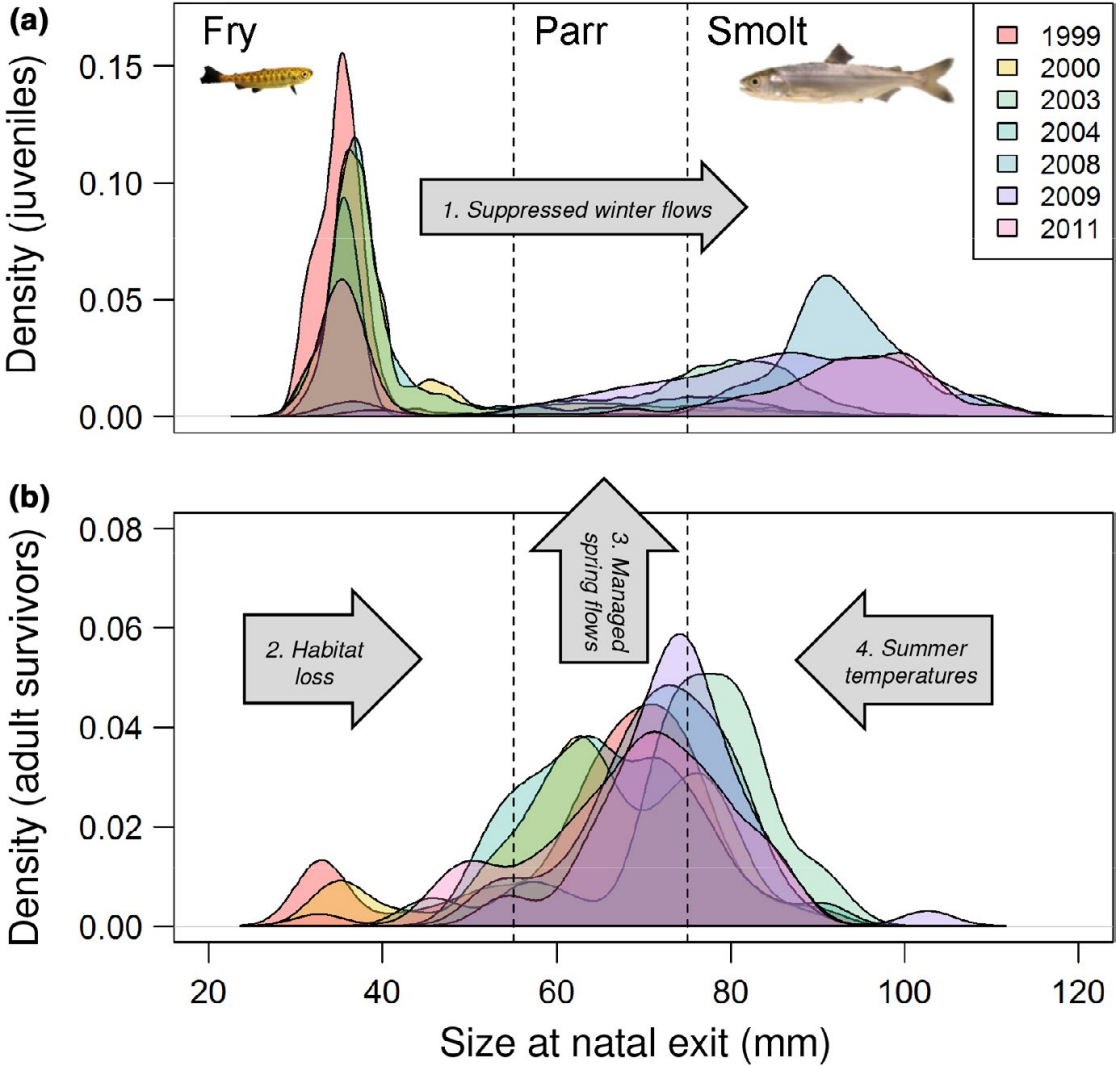
Selection against phenological extremes in a modified riverscape. Natal exit size distributions of (a) juvenile salmon sampled by rotary screw traps, versus (b) surviving adults from the same cohort (reconstructed using otolith strontium isotopes). Suggested hypotheses to explain patterns in trait expression (a) and selection (b) are described in the Discussion and outlined in large arrows: 1. Reduced fry emigration in years with suppressed winter flows (Figure 2d); 2. Fry mortality exacerbated by loss of downstream rearing habitats; 3. Parr benefiting from spring reservoir releases (Figure 2b); 4. Smolt mortality exacerbated by seasonal increases in temperature (and associated changes in water quality and predation rates) along the migratory corridor, which are particularly extreme in the San Joaquin River and southern Delta (Buchanan et al., 2018)

Otolith reconstructions indicated consistent selection against small (early) and large (late) juveniles following emigration from the natal tributary. While juveniles primarily left the natal stream as fry or smolts (Figure 6a), parr migrants were most commonly observed in the surviving adults (38%–60%, mean = 51%, Figure 6b; Table S5). Accordingly, parr exhibited the highest survival rates (Welch ANOVA *F*_2,8.7_ = 4.59, *p* = .044), driven by large differences with early dispersing fry (median = 6.92% vs. 0.20%, respectively; Games‐Howell test *p* = .02; within‐year differences significant in all years except 2009; Figure S7; Table S6). Peak parr emigration in April coincided with managed releases intended to improve downstream survival, and the only month in the salmon emigration period exhibiting inflated (relative to historical) flows (Figure 2b).

Smolts were expected to exhibit the highest survival probabilities based on typical trends in size‐selective mortality and their later emigration timing (i.e., shorter exposure time between natal exit and recruitment). Yet, smolt survival was significantly lower than parr in almost half the years examined, and similar or marginally lower than fry survival in recent years, suggesting both within‐ and among‐year changes in selection (Tables S5 and S6). Potential limitations with our method would be most likely to underestimate the survival rates of newly emerged fry (see Study Limitations), but we are reasonably confident of the survival differences among the larger, later migrating stages. Indeed, focusing on the warm, smolt‐dominated cohort of 2008, there was a clear mode of juveniles emigrating from the natal stream at ca. 90 mm FL, yet most of the adult survivors had already left freshwater by that size (Figure S8), suggesting extremely low survival of the latest migrants (i.e., the smolts).

## 4 DISCUSSION

### 4.1 Altered river flows shaped trait expression and suppressed fry dispersal

While migration timing in juvenile salmonids can be influenced by genetic, maternal, and demographic factors (Cogliati, Unrein, Stewart, Schreck, & Noakes, 2018; Greene & Beechie, 2004), flow is often identified as a key driver (Sykes et al., 2009). Aquatic species in California evolved in a dynamic environment, and various taxa respond to seasonal flow cues, redistributing themselves across the landscape to maximize fitness and growth (Merz, Delaney, Setka, & Workman, 2015; Yarnell et al., 2015). In the Stanislaus River, the natural hydrograph has been chronically flattened, altering physical and ecological processes such as sediment redistribution and migration cues. Today's hydrograph is largely managed in a binary manner—winter peak flows present or absent—resulting in the promotion or suppression of fry dispersal, respectively. Unlike the fry‐dominated portfolios typical for this system (Williams, 2006) and elsewhere (Healey, 1991), fry often comprised fewer than half of the emigrants, even when spawner densities were high (e.g., 2001–2002) and increased density‐dependent migration would be predicted (Greene & Beechie, 2004).

Fry suppression has important implications. Early dispersers can reduce instream competition and spread risk spatially (e.g., use of novel downstream habitats) and temporally (e.g., broadening ocean arrival timings by diversifying growth opportunities) (Phillis et al., 2018). They may also provide a critical role for maintaining genetic diversity (Hamann & Kennedy, 2012). Given seasonal changes in downstream habitat quality selecting against late migrants (Lehman, Huff, Hayes, & Lindley, 2017), and interannual variation in spring transition date at lower latitudes (Spence & Hall, 2010), maintaining a broad migratory window is particularly important for these southernmost populations (Satterthwaite et al., 2014). Furthermore, as discussed below, even marginal improvements to fry survival rates could significantly boost adult recruitment rates. There is considerable uncertainty about the mechanisms controlling emigration timing and the extent to which it represents phenotypic plasticity versus a heritable trait. However, with increasing climate uncertainty on the horizon, strong suppression of any life‐history diversity—whether evolved or plastic—could have serious demographic and evolutionary consequences.

### 4.2 Lower, less variable flows were associated with reduced fish production

Interannual variation in salmon abundance is often linked to fluctuations in ocean conditions (Lindley et al., 2009; Scheuerell & Williams, 2005), but in a warming climate, freshwater flows are playing an increasingly important role (Michel, 2018; Sturrock et al., 2015; Ward, Anderson, Beechie, Pess, & Ford, 2015). Here, recruits‐per‐spawner estimates were often <1 and returns‐per‐spawner were substantially lower (geometric mean = 0.5, Table S5), suggesting negative population growth in the absence of demographic rescue by hatchery strays. Generally, increased flows were accompanied by increased juvenile and adult production, suggesting a flow‐mediated carrying capacity in the natal stream. If reduced winter flows were merely delaying fry emigration (i.e., the lower contributions of fry reflecting extended rearing and emigration at larger sizes), we would expect higher numbers of parr‐ and smolts‐per‐spawner in drier years, but this was not observed. In semi‐arid climates, higher river flows are often associated with higher turbidity and increased water quality, which can interact to reduce predation and physiological stress (Gregory & Levings, 1998; Lehman et al., 2017; Nobriga & Feyrer, 2007). Increased discharge can also stimulate food production and improve access to floodplain and side channel habitats, offering significant growth benefits and predator refugia (Sommer, Nobriga, Harrell, Batham, & Kimmerer, 2001). Importantly, the relationship between rearing flows and cohort strength was still detectable in the adult returns, with higher and more variable flows associated with higher numbers of recruits. The largest deviations from this trend occurred in years characterized by exceptionally poor ocean conditions (Lindley et al., 2009; Michel, 2018; Sturrock et al., 2015), indicating that other factors can still play an important role.

### 4.3 Selection against the tails homogenized the life‐history portfolio

Prevailing ecological theory predicts different phenotypes to be favored under varying environmental conditions (Schindler et al., 2010). Despite significant variation in climate and phenotype expression among years, the optimal emigration strategy did not vary, suggesting strong and consistent selection along the migratory corridor, acting against small, early dispersing fry and large, late migrating smolts. This stabilizing selection was likely driven by density‐dependent mortality of early migrants that has been amplified by the loss of downstream rearing habitats, seasonal, temperature‐related mortality of late migrants, and managed reservoir releases in late spring creating a narrow window of opportunity for parr (Figure 6b).

Historically, the Sacramento‐San Joaquin Delta (Delta) was dominated by floodplains and wetlands that could support large numbers of rearing fry, and provide predator and thermal refugia for smolts (Sommer et al., 2001), but <3% of these habitats remain today (SFEI‐ASC, 2014). Fry were expected to exhibit the lowest survival rates based on their longer exposure period and smaller body size (Sogard, 1997), but they consistently contributed to the adult returns (comprising 5%–23% of adult returns, Table S5). Most reared downstream for multiple weeks, achieving considerable growth in non‐natal habitats before leaving freshwater as smolts (mean freshwater exit size = 77.5 mm ± 10.7 mm *SD*), suggesting a minimum size for successful smoltification. Differences in winter flow management among years were associated with high variability in fry expression, but in fry‐dominated years, their sheer numbers could provide significant demographic boosts if the carrying capacity of downstream habitats were increased, with even marginal changes in survival equating to large differences in recruitment.

In juvenile fishes, selection based on body size typically favors larger, faster growing individuals (Sogard, 1997), yet here, the largest migrants exhibited relatively low survival. We hypothesize that this signals strong time‐selective mortality along a degraded migratory corridor lined with piscivorous fishes, whose metabolic rates are tightly coupled with temperature (Nobriga & Feyrer, 2007). Summer water temperatures in the Delta often exceed 22°C and are accompanied by increased pathogen burden and contaminant loads, which interact to increase physiological stress and reduce predator avoidance capabilities (Lehman et al., 2017). Indeed, smolt survival probabilities were negatively related to mean monthly temperatures in the San Joaquin River just downstream of the natal stream (USGS gauge no. 11303500) during the period of peak smolt migration (*r* = −.75 in April; *r* = −.64 in May). Truncation of the seasonal rearing window caused by warming river temperatures has been identified as a critical issue for Central Valley salmonids (Munsch et al., 2019), but even in more northerly latitudes, seasonal temperature increases have been linked to elevated smolt mortality (Scheuerell et al., 2009).

## 5 STUDY LIMITATIONS

While juveniles typically expressed two main migration pulses, we applied three size classes to try to capture the intermediate size and migratory period. However, the size delineations are somewhat arbitrary and could potentially be decoupled from emigration timing. For example, while the screw trap data showed a strong relationship between emigration date and FL (*r* = .8), there was still considerable spread, and also noise in our FL back‐calculation model (Figure S2). We also could not use otolith daily increments to reconstruct calendar date at habitat transitions, as spawn timing varies on the order of months, and there would be significant error propagation counting daily rings back from the edge of adult otoliths (>2 years). Thus, we could not fully separate the influence of size versus time on emigrant survival, or ascertain whether “parr” primarily represented “fast‐growing fry” or “early‐migrating smolts.”

We also note the potential for otolith ^87^Sr/^86^Sr reconstructions to misidentify fish origin and/or natal exit size. For example, if an individual left the natal stream <14 days (the typical resolution of our ^87^Sr/^86^Sr measurements) after emerging from the gravel, the natal region of the otolith could exhibit the downstream water signature, resulting in the individual potentially being misclassified as a stray. We attempted to reclassify these individuals (see Supplemental Methods), but the survivorship of newly emerged fry may still be underestimated. Additionally, some water samples in the Delta overlapped with the isotopic range of the Stanislaus River (Figure 1). If a juvenile rapidly migrated to one of these locations, the rise in ^87^Sr/^86^Sr associated with movement through the San Joaquin River might not be detectable in their otolith, resulting in overestimation of their natal exit size. We believe that the potential impact on our results is minimal, as the overlapping areas were concentrated in the northern Delta (i.e., off the main emigration route), and because the Delta is tidal and hydrologically dynamic, so these overlapping values were unlikely to remain stable over extended periods of time. Note that both potential sources of error would be most likely to result in underestimated fry survival rates given their earlier emigration dates and longer periods rearing in downstream habitats before entering the ocean.

## 6 MANAGEMENT AND PORTFOLIO EFFECT IMPLICATIONS

Despite consistent selection against early and late migrants, our results revealed that all phenotypes contributed to the reproductive population in every year examined. On average, fry and smolt emigrants produced 1,271 adult recruits per year (48% of overall mean production) and increased recruitment stability by 4%, reducing the CV in adult production from 1.10 (assuming zero fry and smolt survival) to the observed value of 1.06. This marginal effect was largely attributable to smolt migrants, which exhibited the lowest interannual variation in abundance and survival of all the phenotypes. Indeed, in a scenario with only smolts surviving to adulthood, adult production would have been 17% more stable (CV = 0.88) but on average reduced by 70% (1,866 fewer recruits). Conversely, if only fry had survived, production would have been 25% less stable (CV = 1.32) and 81% lower (2,145 fewer recruits). However, given the substantial numbers of fry often produced, even marginal increases in their survival rates would have significant impacts on recruitment. Maintaining fry survival at the levels observed in the latter half of the study (mean of 2008–2011 = 3.56%) would have resulted in 11,872 (2004) to 64,457 (2000) additional recruits from the fry‐dominated cohorts (1999, 2000, 2004, Figure 3), equivalent to 7.3 (1999) to 12.0 (2004) times higher adult production.

Salmon in highly impacted ecosystems often exhibit a weakened portfolio effect, signaled by among‐population synchrony (Griffiths et al., 2014; Schindler et al., 2010); however, empirical data to tease apart the underlying mechanisms are rare. While hatchery strays (Satterthwaite & Carlson, 2015), oceanographic cycles (Kilduff, Di Lorenzo, Botsford, & Teo, 2015), and overharvest of weaker stocks (Griffiths et al., 2014) may also play a role, our data suggest that dam operations and habitat loss should not be overlooked (Beechie, Buhle, Ruckelshaus, Fullerton, & Holsinger, 2006; Moore, McClure, Rogers, & Schindler, 2010). Adjacent watersheds often experience similar climates and manage their dams for similar goals, which could homogenize emigration timings among nearby populations. Shared bottlenecks such as the Sacramento‐San Joaquin Delta could further compress emigration timings, increasing the risk of match‐mismatch events in the ocean (Satterthwaite et al., 2014). Tracking juvenile traits across life stages and habitats can help managers identify mortality hotspots and changes in life‐history diversity through time. As demand for water capture and hydropower grows, it is important to understand and monitor the influence of regulated flows on fish behavior and survival in order to design hydrographs that support healthy ecosystems in a changing climate (Yarnell et al., 2015).

## 7 SUMMARY

Many factors shape trait expression, productivity, and resilience. In an increasingly unpredictable climate, anthropogenic activities that suppress life‐history diversity could have serious consequences, particularly for small boundary populations persisting at ecological and physiological limits (Bridle & Vines, 2007; Herbold et al., 2018). Dams and extreme summer temperatures already restrict the spatiotemporal range of low latitude salmon populations during freshwater residence; trends likely to be exacerbated by future climate change (Munsch et al., 2019). Most climate change models predict reduced future snowpack, impacting water supply reliability, migration cues, and habitat suitability for thermally sensitive species (Cloern et al., 2011). Here, water capture in warm, dry years tended to delay emigration timing when downstream selection against late migrants was the most extreme. This “inversion” of the migratory portfolio increases vulnerability to future droughts, and any compression of the migratory window can erode portfolio effects and increase risk of mismatch with optimal ocean conditions (Satterthwaite et al., 2014; Spence & Hall, 2010). Increasing flow variability over the full migratory period to mimic the natural hydrograph more closely should increase trait diversity and redistribute juveniles across a wider array of habitats. In parallel, augmenting the carrying capacity of natal and downstream habitats (e.g., through increased flow and habitat restoration) should enhance productivity by better supporting phenological extremes. Such measures could also diversify growth opportunities and further broaden ocean arrival timings, buffering recruitment variability through time (Satterthwaite et al., 2014). Further analyses are necessary to disentangle selection mechanisms and explore the cost benefits of managing for resilience (e.g., spreading life histories away from an apparent optimum) versus abundance (e.g., promoting the parr strategy). However, our results suggest that even with this weakened portfolio, maintaining a broad distribution in migration traits still increased mean production and reduced variance. In variable environments facing increasingly volatile futures (Cloern et al., 2011; Swain et al., 2018), it would be prudent to implement actions that promote and maintain diverse life‐history portfolios (Greene et al., 2010; Herbold et al., 2018; Johnson et al., 2014).

## AUTHOR CONTRIBUTIONS

A.S. performed analyses and wrote the manuscript with input from all authors. A.S., R.J., S.C., J.M., J.D., and T.H. developed the work. H.S., S.N., and A.S. performed the modeling.

## DATA AVAILABILITY STATEMENT

The Supporting Data used to generate these results are archived in Dryad (https://doi.org/10.5061/dryad.73n5tb2ss).
