# Supplementary material for: Unnatural selection of salmon life histories in a modified riverscape
Source: Glob Chang Biol. 2019 Dec 2;26(3):1235–47. doi: 10.1111/gcb.14896 (PMC7277499; doi:10.1111/gcb.14896)
Supplement: Supplementary file 1 [file GCB-26-1235-s001.pdf]

Supporting Information

Materials and Methods

Using otolith Sr^{87}/Sr^{86} to estimate provenance and straying rates

Linear discriminant function analysis (LDFA) based on mean natal otolith Sr^{87}/Sr^{86} values was used to predict provenance of the spawning adults using the methods described in (Barnett-Johnson et al. 2008, Sturrock et al. 2015). There were slight alterations to the methods, described below.

Otoliths from known-origin juveniles collected in 1999-2013 and water Sr^{87}/Sr^{86} values collected in 2012-14 (Dataset S1) suggested that the Sr^{87}/Sr^{86} value of the Stanislaus River slightly decreased over the 1999-2011 focus period. Accordingly, we ran two LDFAs using reference samples from early (1999-2002) and late (2011-14) rearing periods to predict provenance of 1999-2003 and 2004-2011 emigrants, respectively. We weighted the models using prior probabilities based on mean hatchery vs. wild contributions to the Stanislaus River spawning grounds in 2010-12 derived from coded wire tag recoveries (Kormos et al. 2012, Palmer-Zwahlen and Kormos 2013, 2015), and assumed that 5% of the natural-origin fraction would comprise wild strays from other streams (Quinn 1993). For tagged hatchery-origin salmon that we analyzed blind, 100% were correctly classified as strays and 97.4% were classified to the correct hatchery-of-origin. Our only misclassification was for a single individual from the Feather River Hatchery (which rarely stray into the Stanislaus River; comprising 1.4-3.5% of strays in 2010-12 according to Kormos et al. 2012, Palmer-Zwahlen and Kormos 2013, 2015), which we incorrectly predicted as being a stray from the Mokelumne Hatchery. Importantly, using jack-knife resampling of the known-origin juveniles used to create the baseline, 100% of Stanislaus River juveniles were correctly assigned. We also used the DFA to predict provenance of water samples collected in the Stanislaus River in 2012-14 (Fig. 1, n=10) and 100% were correctly assigned. As a sensitivity analysis, we repeated the exercise assuming equal priors to all groups and observed <2% difference in the overall proportion of strays vs. returns.

Note, a subset of individuals assigned to the Stanislaus River were *post-hoc* reclassified as strays (5 of 785 individuals) because their otolith profiles exhibited Sr^{87}/Sr^{86} values <0.7060, suggesting Sacramento Basin origin (Fig. 1), or their core patterns were indicative of early dispersal from a different river (Sturrock, unpublished data). Conversely, as detailed below, a subset of individuals assigned to Mokelumne or Feather River Hatcheries were *post-hoc* reclassified as Stanislaus River returns (8 of 785 individuals). If a juvenile emigrated from the Stanislaus River in <14 days of emergence (the typical resolution of a single spot analysis) it could be misclassified as a stray, as the otolith material deposited after exogenous feeding would record the downstream rearing location. The San Joaquin River (SJR, Fig. 1) represents the non-natal rearing location immediately downstream of the Stanislaus River, and the reach immediately below the Stanislaus River overlaps isotopically with both the Feather and Mokelumne River Hatcheries (Dataset 1). Note that the SJR was not included as a potential source in the LDFA as it currently does not support its own fall-run salmon population. In the returning adults, some otolith Sr^{87}/Sr^{86} profiles suggested that the individual had been born in the Stanislaus River, emigrated as ~40mm fry, then reared in the SJR for multiple weeks (an example shown in (Sturrock et al. 2015)). We isolated the individual otolith Sr^{87}/Sr^{86} values for these non-natal rearing periods and treated them as “SJR knowns”. We combined these data with individual measurements across the natal region of otoliths from known-origin Mokelumne and Feather River Hatchery juveniles. For each fish we calculated the mean and SD for the three

"rearing locations", then used these values to train a quadratic DFA (QDFA) assuming equal priors. Using jack-knife resampling, the QDFA predicted 83% of the "SJR rearers" correctly (80% correctly overall). We calculated equivalent metrics for the natal portions of otoliths from all adults that had been initially classified as strays from Mokelumne and Feather River Hatcheries (i.e., the individuals most likely to represent misclassified fry) then used the QDFA to predict their rearing locations. This reclassified 7.6% (23 of 304 individuals) as "SJR rearers". However, given that this is a new approach based on few "knowns" (n=6 adults that we were confident had reared in the SJR, comprising 33 individual spot measurements in putative SJR water), we only accepted the reclassification if all spots were within the range of SJR water values (Dataset S1) and if the otolith microstructure (Barnett-Johnson et al. 2007) also suggested wild origin. As a result, only eight individuals were finally reclassified as Stanislaus River returns that had dispersed as newly emerged fry (~30mm FL) and reared in the SJR.

Using otolith Sr^{87}/Sr^{86} to estimate size at natal and freshwater exit
For returning adults, we reconstructed their size at natal exit using the otolith distance from the core to the last measurement exhibiting a Sr^{87}/Sr^{86} value ± SE within the range of measured values in the otoliths of known-origin juveniles or water samples from the Stanislaus River during a comparable period (see above; Dataset S1). Given that the rotary screw traps are 13.8rkm upstream of the SJR confluence (i.e. the location of the isotopic shift used to identify natal exit in the returning adults, Fig. 1), we used the last recorded "natal value" rather than interpolating among neighboring spots. Size at freshwater exit was estimated as the otolith radius when $^{87}Sr/^{86}Sr$ ratios exceeded the mean value recorded at Chipps Island (0.7077 – see Dataset 1), roughly equivalent to the fish moving into waters >0.5ppt (Hobbs et al. 2010). We used linear interpolation between neighboring spots to estimate the otolith distance at this threshold value (after Phillis et al. 2018). Otolith distance was then converted into fish fork length (FL) using the relationship shown in Fig. S2 (methods detailed in main text).

Adult recruitment
The number of adult recruits produced by each emigration cohort was estimated as estimated escapement (GrandTab; available at www.dfg.ca.gov) corrected for strays and return age distributions, plus ocean and inland harvest. We used year-matched age distributions obtained from adults on the Stanislaus River spawning grounds (CDFW unpublished annual age estimates for unmarked salmon for 2001-2015; Mesick et al. 2009 age estimates for 1996-2000) to estimate the numbers of adult returns per emigration cohort. Annual ocean harvest rates for 3 and 4 year olds were estimated as total ocean harvest divided by the Sacramento Index (Pacific Fishery Management Council 2016). We assumed zero ocean harvest for 2-year old returns (advice of M. O'Farrell, NMFS, as 2 year olds tend to be below minimum size for the commercial fishery. Note that we also ran the analysis assuming equal harvest for all ages and it made little difference to the results). Inland harvest rates were assumed to be 5% each year (after USFWS 2017).

We used two data sources for estimating straying rates into the Stanislaus River: (1) otolith natal assignments from our seven emigration cohorts (see above), and (2) Constant Fractional Marking Program tag recovery data. For (1), as our otolith sample was randomly taken from unmarked fish only, we first removed adclipped spawners from total escapement estimates (querying raw numbers of adclipped fish per year from www.rmpc.org then expanding by the annual mean expansion factor ["estimated_number" field]) before applying our straying

estimates and age frequencies (Table S2). For (2), CFM straying rates were available for 2010-2013 escapements at the time of writing (Kormos et al. 2012, Palmer-Zwahlen and Kormos 2013, 2015, Palmer-Zwahlen et al. 2018). Trucking rates of hatchery fish (a major determinant of straying rate; Sturrock *et al*, 2019) were relatively similar in the years before and after this period, so we applied the first estimate (2010) to preceding escapement years (1997-2009) and the last available estimate (2013) to proceeding years (2014-15). While these recruitment estimates should be treated with caution, in years with coupled with otolith and CFM-derived recruitment estimates (n=7), the overall mean difference in recruitment was 1.5% (Fig. S3).

One should note that weir counts on the Stanislaus River (Peterson et al. 2017) suggest that net upstream passage of adults could be ~35% higher than the Grandtab escapement estimates we used to estimate recruitment (Table S2). As weir data were not available for all years of interest, we used Grandtab data to remain consistent across the whole dataset. It is likely that not all adults passing the weir successfully reach the spawning grounds, but these discrepancies warrant further investigation as they could mean that our juvenile survival probabilities are underestimated. However, we assumed that most error would come from the juvenile passage expansions (note large confidence intervals in Table S4), which we did incorporate into our survival estimates and their associated confidence intervals (Table S5). Importantly, for years with both weir and Grandtab escapement estimates (n=13; 2004-2010, 2012-17), they were highly correlated ($r^2 = 0.92$), suggesting that while absolute recruitment and survival estimates might be slightly underestimated, interannual patterns (e.g. the relationship between rearing flows and recruitment in Fig. 5) would not be altered by updating the underlying dataset.

Testing for differences in phenotype survival probabilities

We compared annual phenotype survival rates using Welch ANOVA and Games-Howell *post-hoc* test as we could not transform the data to meet the assumption of homoscedasticity. Differences in survival within years were estimated by randomly resampling (n=100,000) simulated phenotype survival estimates, i.e. we randomly picked one possible number of recruits for a given phenotype and divided it by one possible number of emigrants of the same phenotype, and repeated the process 100,000 times. For each random sampling, we calculated the difference in survival among phenotypes (e.g. parr S – fry S). The distribution of these values represents the distribution of possible survival differences (equivalent to a t-statistic), with the average difference between them being the expected ("real") difference in survival and the proportion of comparisons exhibiting a difference in survival greater than zero being equivalent to a one-tailed p-value (Manly 2006) (Table S6).

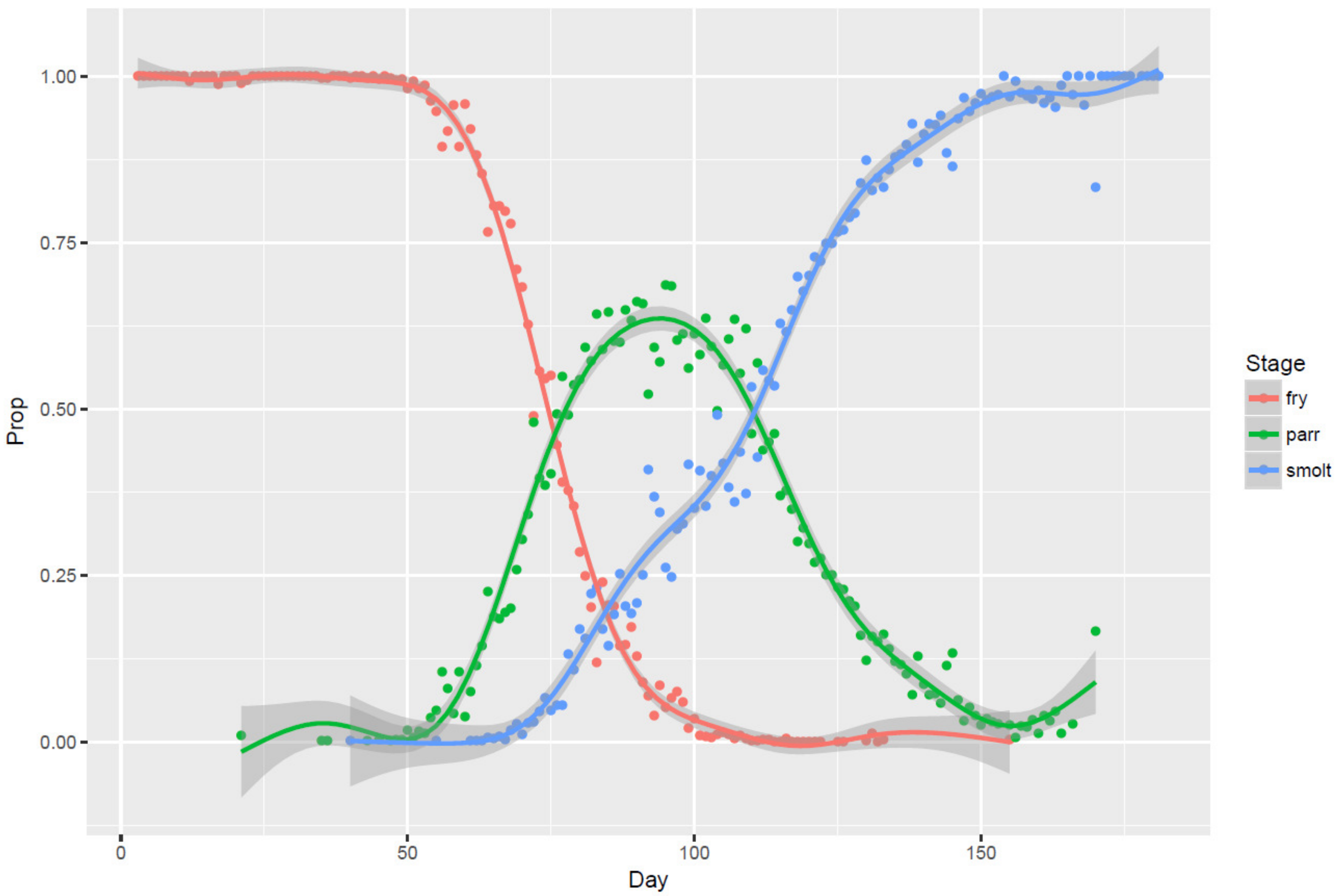

Fig. S1 The proportion of fry, parr, and smolt emigrants (based on expanded rotary screw trap catches) averaged by day for all years with reliable trapping data (1996, 1998-2005, 2006-2014) to demonstrate the general timings that these size classes leave the Stanislaus River. Subtle among-year differences in phenology are shown in Table S3.

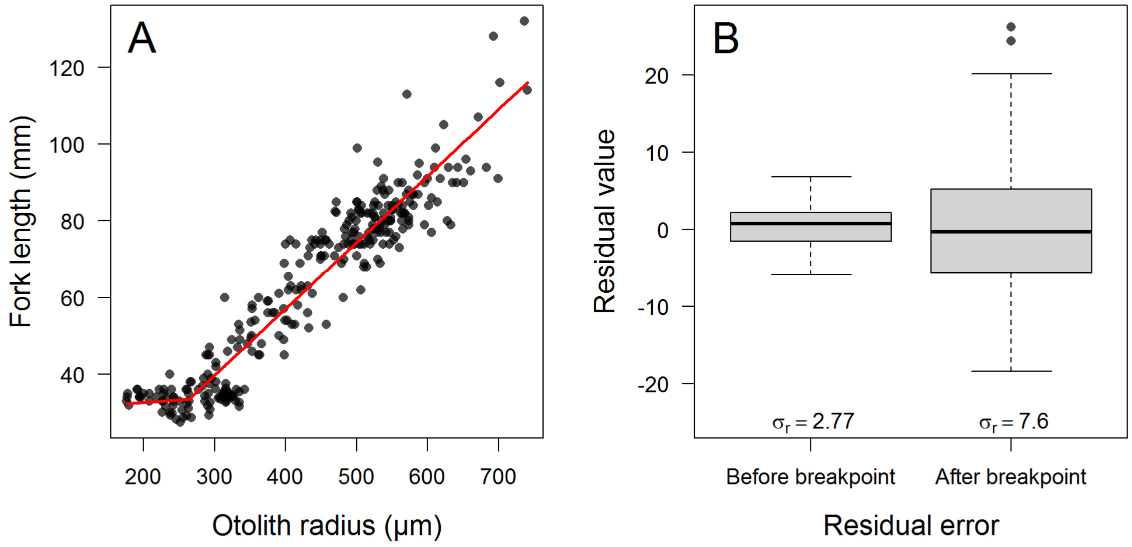

Fig. S2. (A) Broken-stick calibration curve used to reconstruct size at emigration from the otoliths of surviving adults (n=294). (B) The residuals around the line were used to estimate noise around the reconstructed fork lengths and phenotype classifications in the adult recruits.

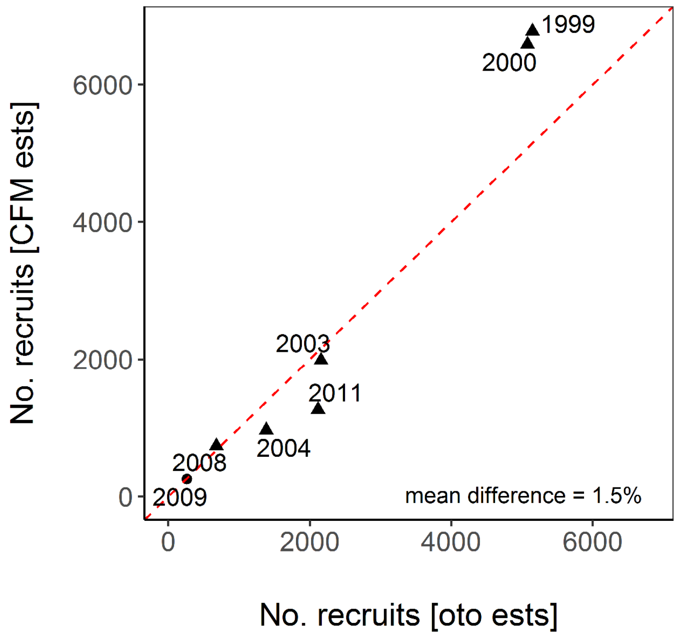

Fig. S3. Difference between recruitment estimates obtained using straying rates based on otolith assignments of unmarked salmon vs. tag recoveries by the Constant Fractional Marking Program (CFM). The 1:1 line is indicated. Points are labeled by emigration year. Of the CFM-based estimates shown, only 2009 (circle) had high certainty (straying rates available for 2, 3 and 4 year old returns). For the other cohorts, we assumed straying rates when CFM data were not available (see Methods above, and Table S2) and thus these recruitment estimates were deemed less reliable, and we always used otolith-derived recruitment estimates where available.

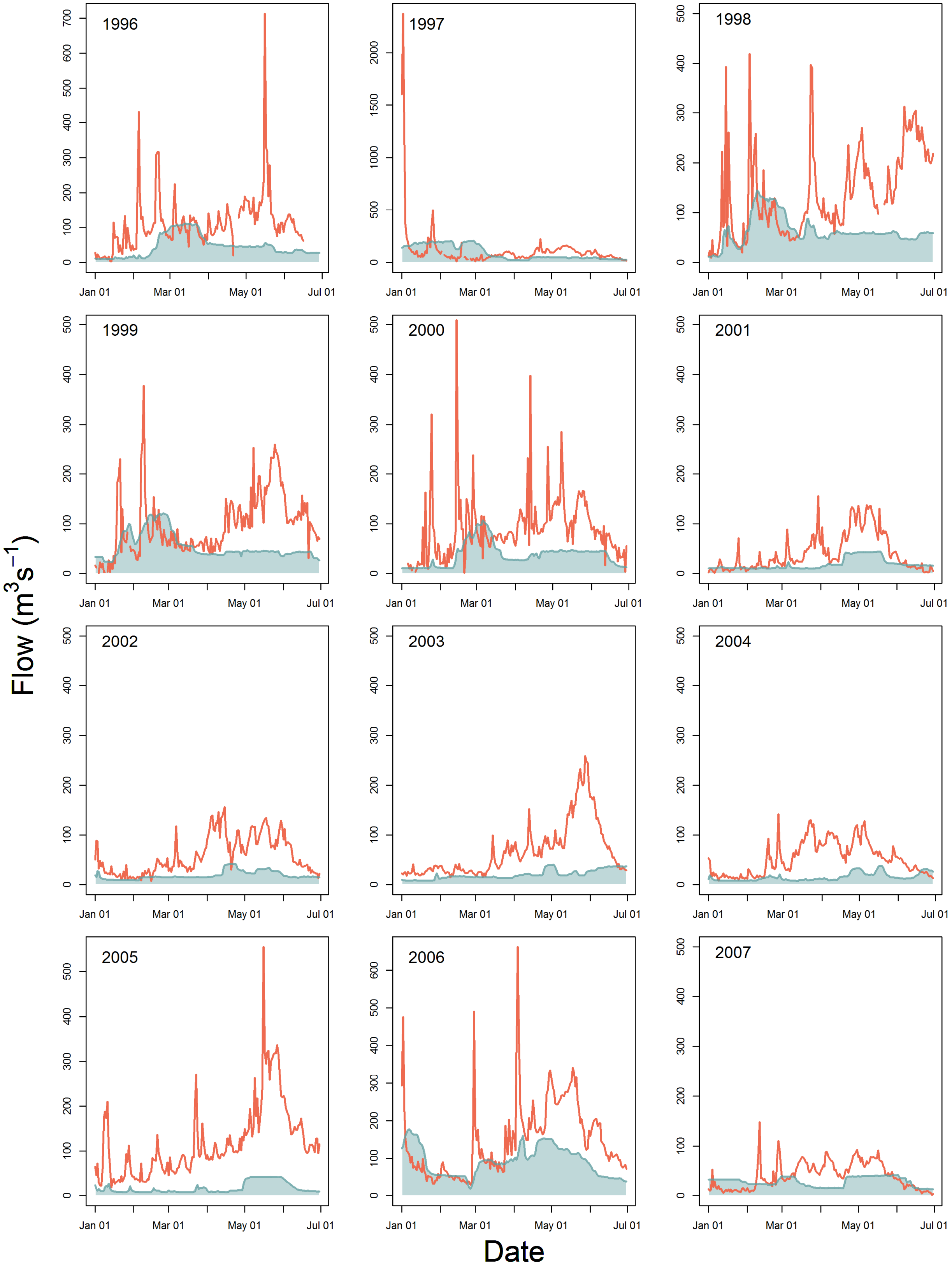

Fig. S4. (continued overleaf)

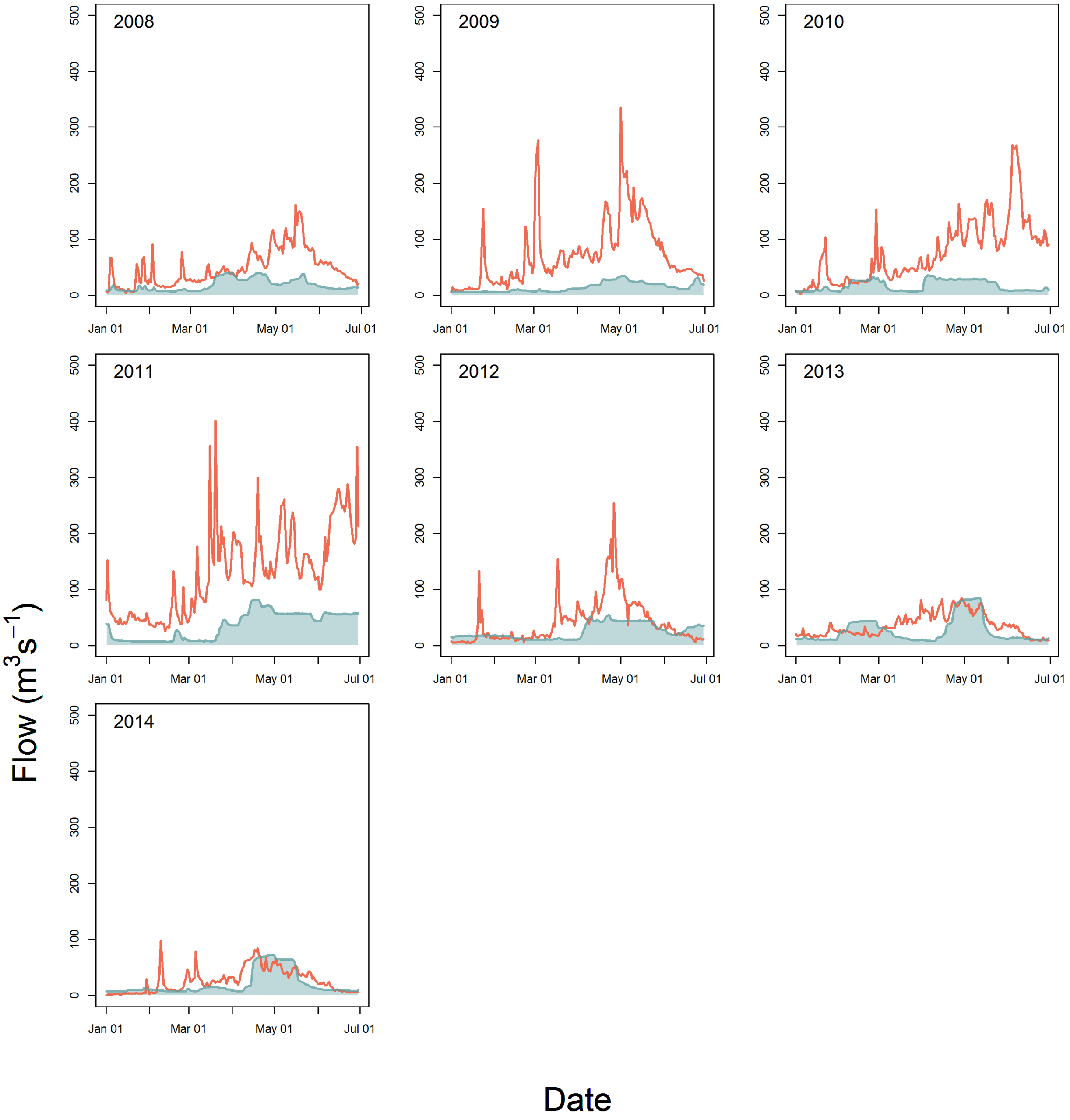

Fig. S4. Unimpaired (red) vs. observed (blue, shaded area) flow in the Stanislaus River for the 19-year study period (1996-2014). Note that high flows in 1997 and 2006 resulted in dangerous operating conditions that precluded the collection of reliable juvenile salmon passage data. Also note different y-axes for wet years 1996-1997 and 2005-2006.

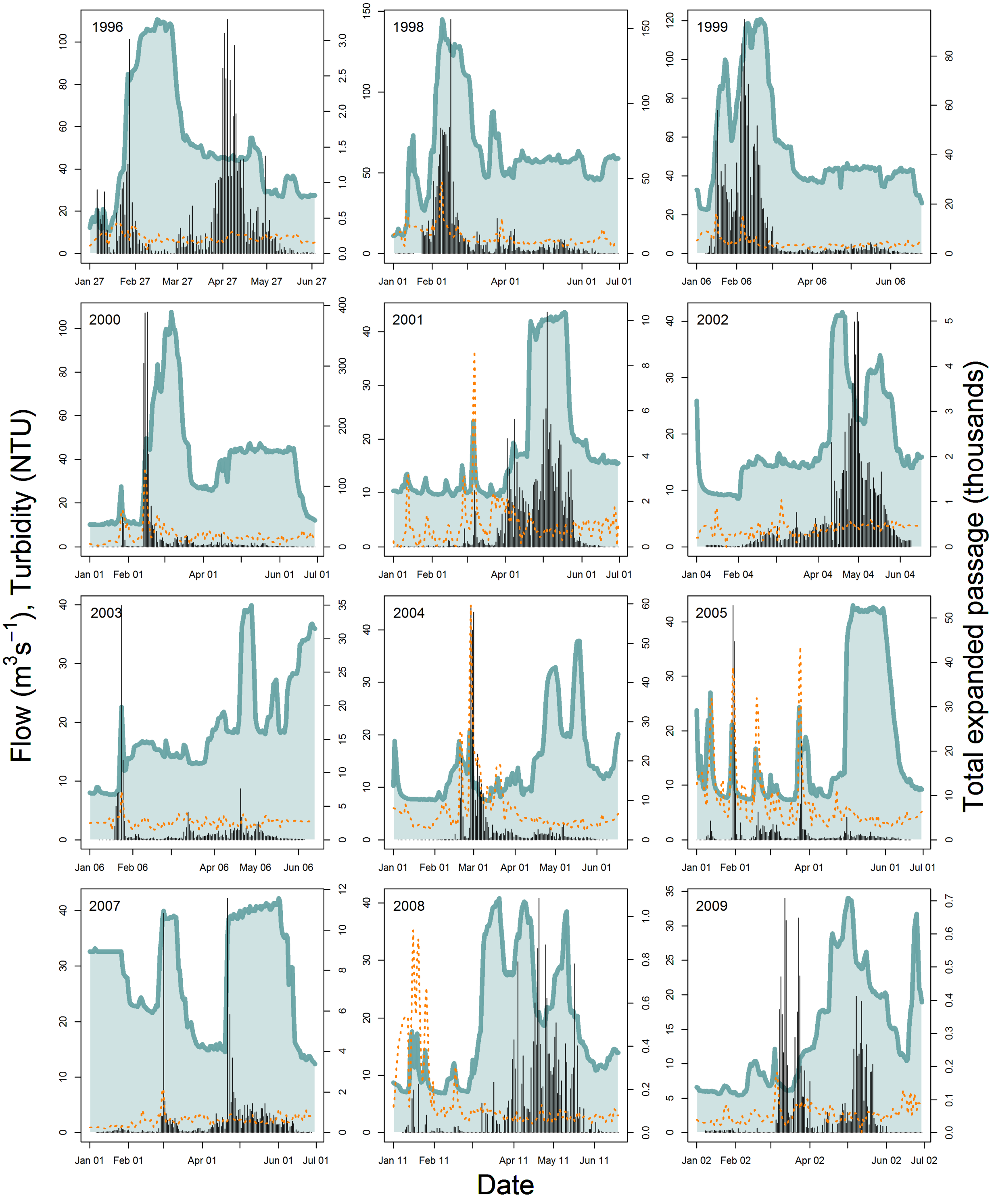

Fig. S5. (continued overleaf)

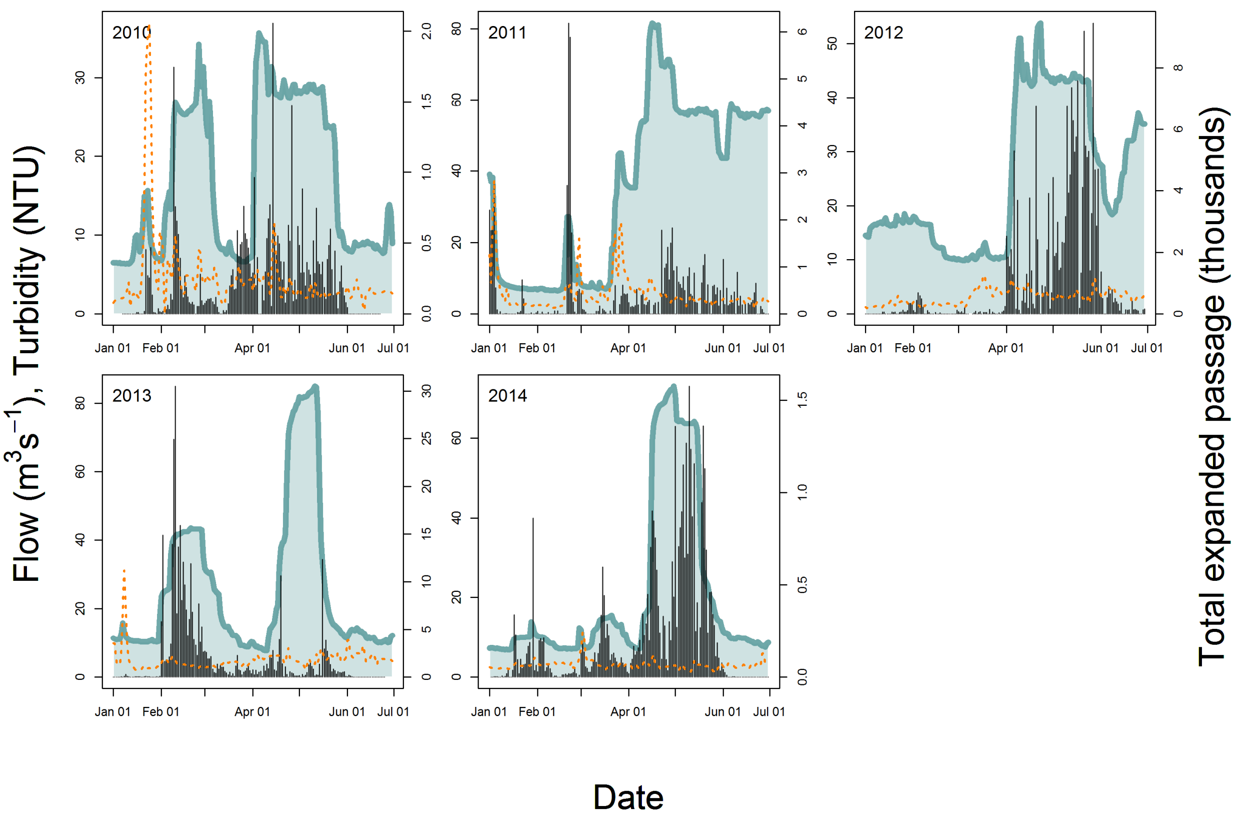

Fig. S5. Total expanded daily juvenile salmon passage (bars) relative to river flow (blue line/shaded area) and turbidity (orange dashed line) for emigration years 1996-2014. Turbidity was measured at the rotary screw trap site using a LaMott turbidity meter (Cramer Fish Sciences 2012). This figure is intended to illustrate migratory responses to flow, not absolute abundance or flows, so y-axes vary substantially among plots. Note that high flows in 1997 and 2006 resulted in dangerous operating conditions that precluded the collection of reliable juvenile salmon passage data.

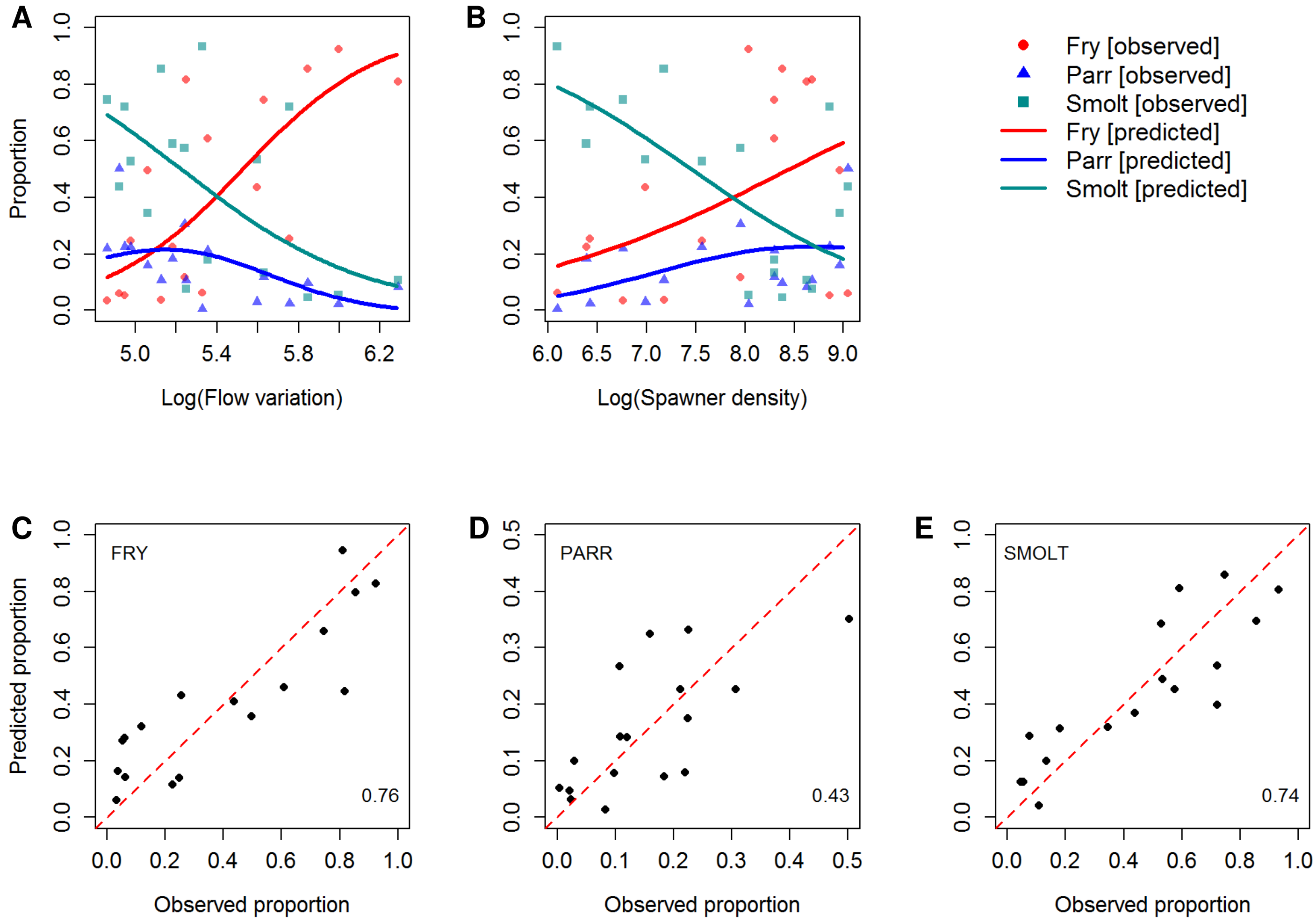

Fig. S6. Controls on juvenile phenotype expression. The proportion of the juvenile salmon that emigrated as fry (circles), parr (triangles) or smolts (squares) is shown as a function of flow variation (A) and spawner density (B); predicted relationships based on the model described in Table 1 are shown as red, blue and green lines, respectively. Plots C to E show observed vs. predicted proportions for each phenotype and the fit (r^2 value) in the bottom-right corner.

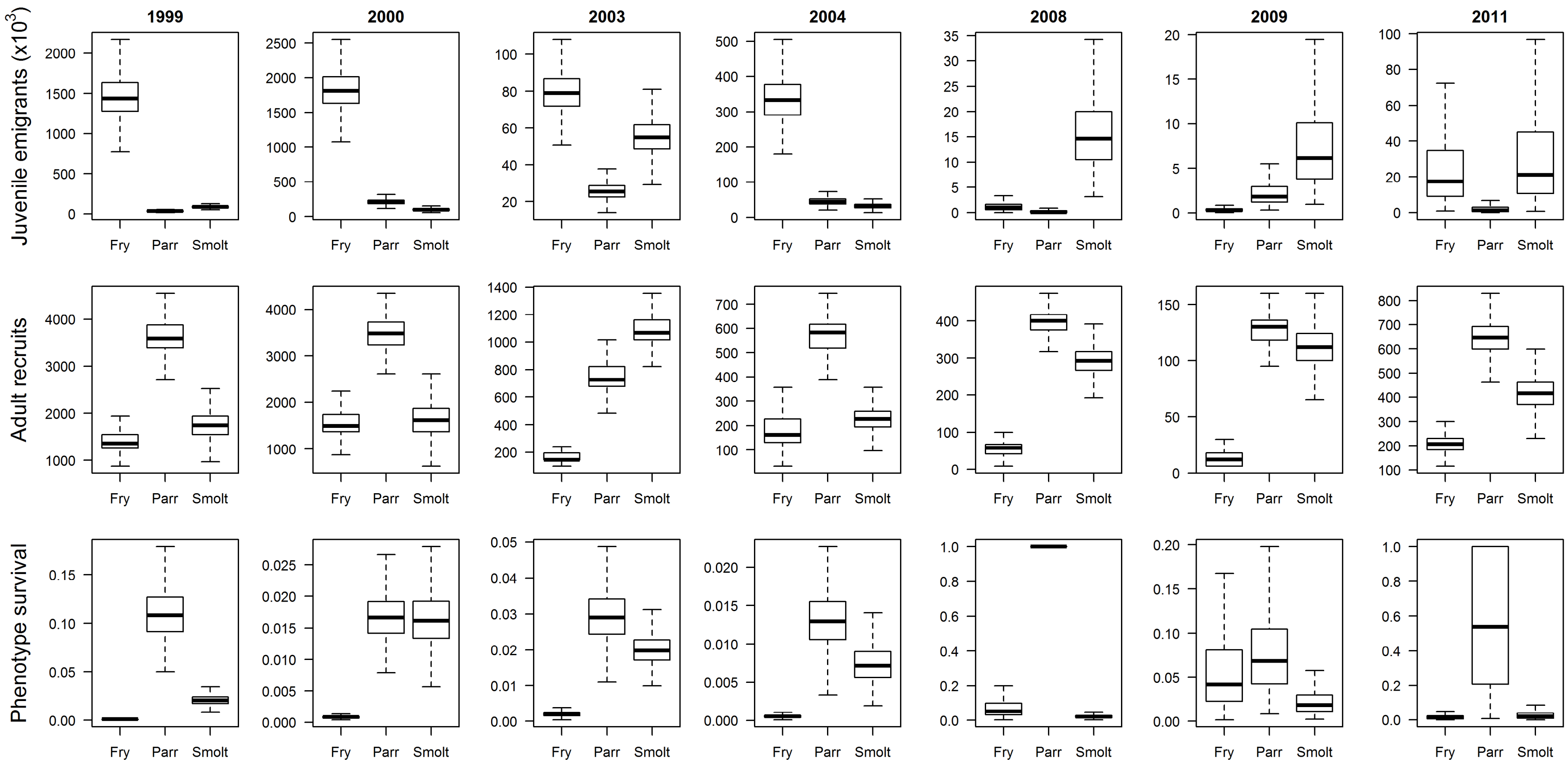

Fig. S7. Annual trends in phenotype abundance, recruitment and survival. Boxplots displaying the abundance of emigrants and adult recruits by phenotype (fry, parr or smolt size at emigration from the natal river) for the seven emigration cohorts with paired otolith reconstructions, and estimated downstream survival of each phenotype (recruits per emigrant). The box represents the interquartile range, the bold line represents the median, and the whiskers represent the 95% confidence interval.

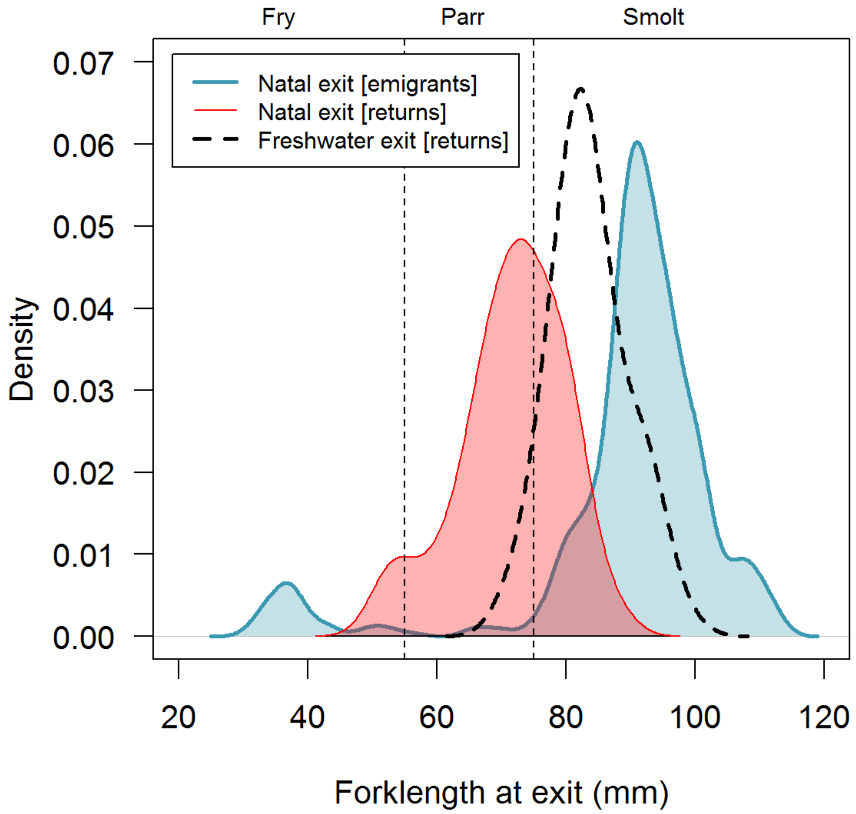

Fig. S8. Density plot showing the size that Stanislaus River origin juveniles emigrated from the natal stream in the smolt-dominated year of 2008 (blue). The size that the adult survivors emigrated from the natal stream (red) then from freshwater (dashed) in 2008 was reconstructed using otolith strontium isotopes and suggests high mortality of the largest (latest) migrants.

Table S1. Sample sizes, age distributions and collection dates of the adult Chinook salmon otoliths analyzed in the current study

Emigration cohort	Escapement year	Age	N	Collection date
1999	2000	2	0	n/a
	2001	3	50	11/29/01 - 12/06/01
	2002	4	43	10/29/02 - 12/12/02
	TOTAL		93 †	
2000	2001	2	6	11/20/01 - 12/06/01
	2002	3	80	10/07/02 - 12/12/02
	2003	4	6	11/12/03 - 12/04/03
	TOTAL		92 §	
2003	2004	2	2	11/08/04 - 11/12/04
	2005	3	56	11/02/05 - 12/15/05
	2006	4	25	11/15/06 - 12/06/06
	TOTAL		83	
2004	2005	2	4	11/08/05 - 12/14/05
	2006	3	75	10/11/06 - 12/12/06
	2007	4	3	11/27/07 - 12/17/07
	TOTAL		82	
2008	2009	2	23	10/29/09 - 12/14/09
	2010	3	95	11/03/10 - 12/16/10
	2011	4	14	11/15/11 - 12/13/11
	TOTAL		132 ‡	
2009	2010	2	17	11/03/10 - 11/24/10
	2011	3	83	10/24/11 - 12/13/11
	2012	4	3	10/30/12 - 11/06/12
	TOTAL		103	
2011	2012	2	40	10/30/12 - 12/12/12
	2013	3	160	10/29/13 - 12/04/13
	2014	4	0	n/a
	TOTAL		200††	
	GRAND TOTAL		785	

† Includes 1 CWT fish analyzed blind to validate natal assignment accuracy.
§ Includes 27 CWT fish analyzed blind to validate natal assignment accuracy.
‡ Includes 1 CWT fish analyzed blind to validate natal assignment accuracy.
†† Includes 7 CWT fish analyzed blind to validate natal assignment accuracy.

Table S2. Data used to estimate the number of adult recruits produced by each emigration cohort (primarily represented by 2, 3, and 4 year old returns). Table continues overleaf (where footnotes include all data sources). We used Constant Fractional Marking Program (CFM) and otolith (oto) derived straying rates, estimated for all fish (by escapement year) and for unmarked spawners (by emigration cohort), respectively. For years prior to CFM we applied the earliest available stray rate, and for years after the last available report we applied the last available estimate (asterisks). As such, some caution should be applied to all CFM recruitment estimates except emigration cohorts 2008 and 2009 (which had published straying rates for age 2, 3, and 4 spawners available).

Return year	No. spawners [‡]	No. adclip [§]	Prop. strays [CFM]	CFM source	Emig-ration year	Return age (yrs) [¶]	Age prop. [unmarked spawners]	No. returns [CFM]	Prop. unmarked strays (oto)	No. returns (oto)	Ocean harvest rate [†]	Inland harvest rate [¥]	No. recruits (CFM)	No. recruits (oto)
1997	5588		0.50	[1] *	1996	2	0.12	335			0.00	0.05	353	
1997	5588		0.50	[1] *	1995	3	0.81	2263			0.66	0.05	7007	
1997	5588		0.50	[1] *	1994	4	0.06	168			0.66	0.05	519	
1997	5588		0.50	[1] *	1993	5	0.00	0			0.66	0.05	0	
1998	3087		0.50	[1] *	1997	2	0.43	664			0.00	0.05	699	
1998	3087		0.50	[1] *	1996	3	0.29	448			0.56	0.05	1071	
1998	3087		0.50	[1] *	1995	4	0.28	432			0.56	0.05	1034	
1998	3087		0.50	[1] *	1994	5	0.00	0			0.56	0.05	0	
1999	4349		0.50	[1] *	1998	2	0.26	565			0.00	0.05	595	
1999	4349		0.50	[1] *	1997	3	0.67	1457			0.44	0.05	2739	
1999	4349		0.50	[1] *	1996	4	0.07	152			0.44	0.05	286	
1999	4349		0.50	[1] *	1995	5	0.00	0			0.44	0.05	0	
2000	8498	81	0.50	[1] *	1999	2	0.06	255	0.24	384	0.00	0.05	268	404
2000	8498	81	0.50	[1] *	1998	3	0.68	2889			0.55	0.05	6759	
2000	8498	81	0.50	[1] *	1997	4	0.25	1062			0.55	0.05	2485	
2000	8498	81	0.50	[1] *	1996	5	0.00	0			0.55	0.05	0	
2001	7033	747	0.50	[1] *	2000	2	0.04	140	0.18	203	0.00	0.05	147	214
2001	7033	747	0.50	[1] *	1999	3	0.49	1730	0.24	2353	0.35	0.05	2802	3811
2001	7033	747	0.50	[1] *	1998	4	0.47	1647			0.35	0.05	2667	
2002	7787	1471	0.50	[1] *	2001	2	0.20	775			0.00	0.05	816	
2002	7787	1471	0.50	[1] *	2000	3	0.50	1956	0.18	2588	0.43	0.05	3613	4779
2002	7787	1471	0.50	[1] *	1999	4	0.29	1123	0.24	1387	0.43	0.05	2075	2561
2002	7787	1471	0.50	[1] *	1998	5	0.01	39			0.43	0.05	72	
2003	5902	1532	0.50	[1] *	2002	2	0.15	442			0.00	0.05	465	
2003	5902	1532	0.50	[1] *	2001	3	0.64	1894			0.50	0.05	3987	
2003	5902	1532	0.50	[1] *	2000	4	0.21	615	0.18	743	0.50	0.05	1295	1564
2004	4015	201	0.50	[1] *	2003	2	0.32	643	0.51	604	0.00	0.05	677	635
2004	4015	201	0.50	[1] *	2002	3	0.51	1020			0.71	0.05	3704	
2004	4015	201	0.50	[1] *	2001	4	0.17	338			0.71	0.05	1228	
2004	4015	201	0.50	[1] *	2000	5	0.00	6	0.18	9	0.71	0.05	20	31
2005	1427	132	0.50	[1] *	2004	2	0.14	98	0.63	65	0.00	0.05	103	69
2005	1427	132	0.50	[1] *	2003	3	0.67	477	0.51	428	0.52	0.05	1047	938
2005	1427	132	0.50	[1] *	2002	4	0.19	136			0.52	0.05	299	
2005	1427	132	0.50	[1] *	2001	5	0.00	2			0.52	0.05	3	
2006	1923	74	0.50	[1] *	2005	2	0.12	115			0.00	0.05	121	
2006	1923	74	0.50	[1] *	2004	3	0.64	619	0.63	436	0.45	0.05	1185	834
2006	1923	74	0.50	[1] *	2003	4	0.24	227	0.51	216	0.45	0.05	434	412
2007	443	0	0.50	[1] *	2006	2	0.25	55			0.00	0.05	58	
2007	443	0	0.50	[1] *	2005	3	0.58	129			0.59	0.05	332	
2007	443	0	0.50	[1] *	2004	4	0.17	37	0.63	27	0.59	0.05	95	69
2008	865	48	0.50	[1] *	2007	2	0.09	38			0.00	0.05	40	
2008	865	48	0.50	[1] *	2006	3	0.88	382			0.06	0.05	428	
2008	865	48	0.50	[1] *	2005	4	0.03	13			0.06	0.05	14	
2009	595	86	0.50	[1] *	2008	2	0.30	88	0.31	104	0.00	0.05	93	109
2009	595	86	0.50	[1] *	2007	3	0.43	129			0.01	0.05	137	
2009	595	86	0.50	[1] *	2006	4	0.27	81			0.01	0.05	86	
2010	1086	266	0.50	[1]	2009	2	0.16	89	0.58	56	0.00	0.05	94	59
2010	1086	266	0.50	[1]	2008	3	0.82	445	0.31	461	0.16	0.05	557	578
2010	1086	266	0.50	[1]	2007	4	0.02	9			0.16	0.05	12	
2011	1309	664	0.83	[2]	2010	2	0.48	108			0.00	0.05	113	
2011	1309	664	0.83	[2]	2009	3	0.44	98	0.58	118	0.34	0.05	156	188
2011	1309	664	0.83	[2]	2008	4	0.08	17	0.31	34	0.34	0.05	27	55
2012	4006	3192	0.83	[3]	2011	2	0.28	193	0.71	67	0.00	0.05	204	71
2012	4006	3192	0.83	[3]	2010	3	0.70	480			0.44	0.05	902	
2012	4006	3192	0.83	[3]	2009	4	0.01	8	0.58	4	0.44	0.05	15	7

2013	2845	799	0.66	[4]	2012	2	0.10	97			0.00	0.05	102	
2013	2845	799	0.66	[4]	2011	3	0.85	819	0.71	503	0.47	0.05	1627	998
2013	2845	799	0.66	[4]	2010	4	0.05	51			0.47	0.05	102	
2014	3060	554	0.66	[4] *	2013	2	0.36	371			0.00	0.05	390	
2014	3060	554	0.66	[4] *	2012	3	0.53	548			0.55	0.05	1281	
2014	3060	554	0.66	[4] *	2011	4	0.12	122	0.71	85	0.55	0.05	285	199
2015	6136		0.66	[4] *	2014	2	0.32	667			0.00	0.05	702	
2015	6136		0.66	[4] *	2013	3	0.62	1301			0.49	0.05	2685	
2015	6136		0.66	[4] *	2012	4	0.06	119			0.49	0.05	245	

General data sources:

‡ GrandTab (http://www.dfg.ca.gov/fish/Resources/Chinook/CValleyAssessment.asp)

§ Regional Mark Processing Center (http://www.rmpc.org). Expansion factors missing in 2000 - applied mean for all other years.

¶ Source: CDFW La Grange California - unpublished scale read data for unmarked spawners on the Stanislaus River natural spawning grounds (2001-2015). For years prior to 2001, we used age distributions provided in “Mesick, Marston and Heyne (2009). Estimating recruitment for fall-run Chinook salmon populations in the Stanislaus, Tuolumne, and Merced Rivers [Table 16] California State Water Resources Control Board: Delta Flow Criteria Informational Proceeding” available at: www.waterboards.ca.gov/waterrights/water_issues/programs/bay_delta/deltaflow/docs/exhibits/cspa/cspa_exh13.pdf

† Pacific Fishery Management Council (2016) Preseason Report I: Stock Abundance Analysis and Environmental Assessment Part 1 for 2016 Ocean Salmon Fishery Regulations. Assumed zero harvest on age 2 returns (based on Michael O’Farrell, NMFS, NOAA, 2017 pers. comm.)

¥ USFWS “Chinookprod” database. Unpublished database maintained by the Anadromous Fish Restoration Program and Comprehensive Assessment and Monitoring Program, Lodi and Sacramento, California.

Sources for the CFM stray rates:

[1] Kormos, B., Palmer-Zwahlen, M., & Low, A. (2012). Recovery of coded-wire tags from Chinook salmon in California's Central Valley Escapement and Ocean Harvest in 2010. Retrieved from Fisheries Branch Administrative Report 2012-02. Retrieved from: http://cahatcheryreview.com/bibliography/: http://cahatcheryreview.com/bibliography/

[2] Palmer-Zwahlen, M., & Kormos, B. (2013). Recovery of coded-wire tags from Chinook salmon in California's Central Valley Escapement and Ocean Harvest in 2011. Retrieved from https://nrm.dfg.ca.gov/FileHandler.ashx?DocumentID=75609

[3] Palmer-Zwahlen, M., & Kormos, B. (2015). Recovery of Coded-Wire Tags from Chinook Salmon in California’s Central Valley Escapement, Inland Harvest, and Ocean Harvest in 2012. Retrieved from https://nrm.dfg.ca.gov/FileHandler.ashx?DocumentID=112524

[4] Palmer-Zwahlen, M., Gusman, V., & Kormos, B. (2018). Recovery of Coded-Wire Tags from Chinook Salmon in California’s Central Valley Escapement, Inland Harvest, and Ocean Harvest in 2013. Retrieved from https://nrm.dfg.ca.gov/FileHandler.ashx?DocumentID=162355

Table S3. Phenology of juvenile Chinook salmon migratory phenotypes emigrating from the Stanislaus River between 1996 and 2014 (excluding 1997 and 2006 due to trap issues). The peak migratory period and peak date were calculated as the interquartile range and median date at emigration, respectively.

**Year**	**All stages**		**Fry (≤55mm FL)**			**Parr (55-75mm FL)**			**Smolts (>75mm FL)**		
	**Mean date (±SD)**	**Mean FL (±SD)**	**Full range**	**Peak period**	**Peak date**	**Full range**	**Peak period**	**Peak date**	**Full range**	**Peak period**	**Peak date**
1996	Apr 13 (36)	84.3 (19.1)	71 (Feb 1-Apr 12)	15 (Feb 8-Feb 23)	Feb 20	100 (Feb 16-May 26)	26 (Mar 27-Apr 22)	Apr 5	144 (Feb 4-Jun 27)	12 (Apr 27-May 9)	May 2
1998	Feb 25 (30.7)	49.2 (19.1)	116 (Jan 3-Apr 28)	9 (Feb 7-Feb 16)	Feb 12	97 (Feb 18-May 25)	14 (Mar 24-Apr 7)	Mar 31	116 (Mar 5-Jun 29)	22 (Apr 26-May 18)	May 11
1999	Feb 17 (26.7)	38.4 (11.5)	142 (Jan 13-Jun 3)	17 (Feb 2-Feb 19)	Feb 12	119 (Feb 14-Jun 12)	32 (Mar 31-May 2)	Apr 16	116 (Mar 5-Jun 29)	23 (May 5-May 28)	May 18
2000	Feb 24 (24.1)	42.5 (12.3)	114 (Jan 2-Apr 25)	3 (Feb 14-Feb 17)	Feb 16	115 (Feb 4-May 29)	28 (Mar 18-Apr 15)	Apr 1	109 (Mar 8-Jun 25)	33 (Apr 15-May 18)	May 9
2001	Apr 24 (20.6)	72.2 (9.7)	132 (Jan 1-May 12)	8 (Mar 2-Mar 10)	Mar 6	95 (Mar 6-Jun 9)	25 (Apr 7-May 2)	Apr 19	107 (Mar 1-Jun 16)	13 (May 2-May 15)	May 8
2002	Apr 24 (23.5)	78.7 (8.2)	80 (Jan 11-Mar 31)	12 (Feb 17-Feb 1)	Feb 24	122 (Feb 9-Jun 10)	18 (Apr 15-May 3)	Apr 28	103 (Feb 1-Jun 11)	18 (Apr 23-May 11)	May 1
2003	Mar 9 (43.2)	57.7 (23.1)	79 (Jan 23-Apr 11)	3 (Jan 27-Jan 30)	Jan 29	117 (Feb 5-Jun 1)	26 (Mar 17-Apr 12)	Mar 20	106 (Feb 24-Jun 9)	20 (Apr 17-May 7)	Apr 24
2004	Mar 10 (21.8)	44.8 (15.5)	89 (Jan 19-Apr 17)	5 (Feb 28-Mar 4)	Mar 1	95 (Feb 26-May 31)	26 (Mar 25-Apr 20)	Apr 4	96 (Mar 4-Jun 8)	14 (Apr 26-May 10)	May 5
2005	Mar 1 (39.3)	51.4 (19.3)	101 (Jan 1-Apr 11)	6 (Jan 30-Feb 5)	Jan 31	117 (Feb 14-Jun 10)	11 (Mar 14-Mar 25)	Mar 25	98 (Mar 14-Jun 20)	25 (Apr 24-May 19)	May 4
2007	Apr 17 (33.5)	67.7 (19.9)	126 (Jan 7-May 12)	2 (Feb 1-Mar 2)	Feb 1	106 (Mar 9-Jun 23)	5 (Apr 20-Apr 25)	Apr 20	105 (Mar 13-Jun 26)	27 (Apr 23-May 20)	May 6
2008	Apr 27 (27)	89 (14.2)	71 (Jan 20-Mar 31)	52 (Jan 26-Mar 18)	Jan 30	63 (Feb 1-May 2)	18 (Mar 22-Apr 9)	Apr 7	90 (Mar 18-Jun 16)	18 (Apr 26-May 14)	May 5
2009	Apr 7 (29.3)	84.5 (13.5)	84 (Jan 9-Apr 2)	40 (Jan 28-Mar 8)	Mar 5	60 (Mar 7-May 6)	3 (Mar 10-Mar 13)	Mar 12	86 (Mar 7-Jun 1)	50 (Mar 23-May 12)	Apr 27
2010	Apr 4 (34.6)	72.1 (23.8)	121 (Jan 11-May 11)	7 (Feb 9-Feb 16)	Feb 10	70 (Mar 2-May 11)	18 (Mar 23-Apr 10)	Mar 28	112 (Feb 9-May 31)	28 (Apr 13-May 11)	Apr 25
2011	Mar 29 (51.3)	69.9 (29.3)	129 (Jan 1-May 9)	49 (Jan 4-Feb 22)	Feb 21	77 (Feb 14-May 1)	11 (Mar 26-Apr 6)	Mar 29	116 (Mar 2-Jun 26)	30 (Apr 25-May 25)	May 6
2012	May 6 (24)	81.4 (9.9)	120 (Jan 12-May 11)	43 (Jan 31-Mar 14)	Feb 5	91 (Mar 12-Jun 11)	35 (Apr 6-May 11)	Apr 16	118 (Mar 3-Jun 29)	19 (May 4-May 23)	May 15
2013	Mar 3 (33.9)	45.3 (17)	108 (Jan 1-Apr 18)	11 (Feb 9-Feb 20)	Feb 13	102 (Feb 22-Jun 3)	21 (Mar 28-Apr 18)	Apr 12	94 (Mar 1-Jun 3)	10 (May 7-May 17)	May 15
2014	Apr 18 (34.1)	72.9 (14)	127 (Jan 4-May 10)	11 (Jan 25-Feb 5)	Jan 29	132 (Jan 21-Jun 1)	31 (Apr 7-May 8)	Apr 23	92 (Mar 7-Jun 7)	22 (Apr 22-May 14)	May 6
**Mean**	**Mar 28 (31.4)**	**64.8 (16.4)**	**106 (Jan 10-Apr 25)**	**17 (Feb 6-Feb 23)**	**Feb 15**	**99 (Feb 20-May 28)**	**20 (Mar 27-Apr 17)**	**Apr 6**	**106 (Mar 2-Jun 16)**	**23 (Apr 23-May 15)**	**May 5**

Table S4. Abundance (median ± 95% confidence intervals) and proportions of juvenile Chinook salmon migratory phenotypes emigrating from the Stanislaus River between 1996 and 2014 based on expanded catches from rotary screw traps (excluding 1997 and 2006 due to trap issues).

Emigration year	Number of emigrants: Median [proportion] (95%CI)			
	Total	Fry (≤55mm FL)	Parr (>55 to ≤75mm FL)	Smolt (>75mm FL)
1996	65,573 (50,028-85,755)	16,662 [26%] (11,239-24,648)	1,542 [2%] (453-3,542)	47,122 [72%] (35,204-63,225)
1998	1,788,696 (1,196,901-2,712,815)	1,449,763 [81%] (945,928-2,196,562)	148,099 [8%] (94,704-241,464)	193,673 [11%] (130,156-292,127)
1999	1,556,320 (1,112,018-2,255,534)	1,438,927 [92%] (1,016,190-2,100,633)	32,973 [2%] (21,932-51,125)	84,526 [5%] (60,284-118,350)
2000	2,120,223 (1,577,379-2,915,064)	1,809,794 [86%] (1,337,351-2,495,523)	207,303 [10%] (141,238-310,174)	99,382 [5%] (70,180-145,793)
2001	174,693 (134,492-225,943)	10,499 [6%] (8,151-13,434)	87,227 [50%] (64,217-120,121)	76,094 [44%] (56,013-104,092)
2002	96,584 (71,645-130,565)	5,140 [5%] (3,087-8,044)	21,704 [23%] (14,544-33,479)	68,718 [72%] (49,785-96,048)
2003	159,715 (120,133-210,360)	78,994 [50%] (59,795-103,916)	25,378 [16%] (17,889-36,282)	54,881 [34%] (38,416-76,288)
2004	409,522 (280,464-587,686)	333,337 [81%] (227,735-474,741)	43,765 [11%] (27,420-69,814)	34,094 [8%] (20,596-54,917)
2005	266,356 (200,691-364,781)	161,151 [61%] (123,406-216,983)	56,187 [21%] (38,449-82,662)	47,885 [18%] (31,457-73,873)
2007	75,661 (25,760-264,747)	18,751 [25%] (6,647-63,579)	16,947 [22%] (5,671-62,034)	39,883 [53%] (13,054-139,851)
2008	16,046 (6,196-39,913)	977 [6%] (125-4,205)	67 [0%] (0-1,880)	14,405 [93%] (5,605-36,841)
2009	8,313 (2,549-34,474)	280 [3%] (83-1,277)	1,817 [22%] (582-7,377)	6,120 [74%] (1,839-25,586)
2010	25,529 (4,885-143,876)	5,731 [23%] (1,098-34,478)	4,644 [18%] (928-28,900)	14,868 [59%] (2,661-93,038)
2011	40,414 (5,955-314,635)	17,332 [44%] (2,644-128,433)	1,168 [3%] (53-15,618)	21,235 [53%] (2,956-174,427)
2012	159,005 (54,300-478,185)	5,754 [4%] (1,868-19,963)	17,099 [11%] (5,054-57,969)	136,267 [86%] (44,698-401,919)
2013	326,669 (233,094-477,059)	242,870 [75%] (168,942-364,803)	38,935 [12%] (26,341-59,855)	43,853 [13%] (30,398-64,780)
2014	36,535 (22,991-56,514)	4,296 [12%] (2,712-6,971)	11,124 [31%] (6,326-19,541)	20,700 [57%] (12,695-34,742)

Table S5. Juvenile salmon survival between emigration from the natal stream and adult recruitment in 1996-2014. For years with paired otolith reconstructions we estimate the contribution, abundance and survival probabilities of fry, parr and smolt emigrants. Adult 'returns' represent the unmarked escapement corrected for hatchery strays and return age (Table S2); 'recruits' represent returns plus harvest. Abundance of juvenile emigrants was estimated from expanded rotary screw trap catches (Table S4). Noise in the otolith size-fork length relationship (Fig. S2) was used to generate 95% confidence intervals around phenotype contributions to the escapement and the number of recruits (in parentheses). Confidence intervals around phenotype survival probabilities incorporated this error term along with sampling (catch) and estimation (efficiency model) error. The data used to estimate the phenotype survival rates in this table are visualized in Fig. S7. Parr survival in 2008 § was assumed to be anomalously high as a result of low catches resulting in uncertain juvenile passage estimates (n=4 caught the entire season).

Emigration year	No. recruits	No. returns	Emigrants per spawner	Recruits per spawner	Returns per spawner	Emigrant survival (%)	Fry recruits (≤55mm FL)			Parr recruits (>55 to <75mm FL)			Smolt recruits (>75mm FL)		
							Contribution	Abundance	Survival (%)	Contribution	Abundance	Survival (%)	Contribution	Abundance	Survival (%)
1996	1,710	935	105.6	2.76	1.51	2.62% (1.99-3.43%)									
1997	5,922	3,183		35.25	18.95										
1998	10,092	5,140	320.7	1.81	0.92	0.56% (0.37-0.84%)									
1999	6,777	4,124	504.0	2.20	1.34	0.44% (0.30-0.61%)	21% (16-26%)	1,452 (1,065-1,743)	0.10% (0.06-0.15%)	53% (43-63%)	3,582 (2,904-4,260)	10.87% (6.67-16.82%)	26% (19-34%)	1,743 (1,259-2,323)	2.07% (1.29-3.23%)
2000	6,588	3,543	488.0	1.51	0.81	0.31% (0.23-0.42%)	23% (15-30%)	1,492 (994-1,989)	0.08% (0.05-0.13%)	53% (42-64%)	3,481 (2,735-4,226)	1.66% (1.05-2.57%)	25% (17-32%)	1,616 (1,119-2,113)	1.63% (0.94-2.57%)
2001	6,034	3,008	20.6	0.71	0.35	3.45% (2.67-4.51%)									
2002	4,468	1,599	13.7	0.64	0.23	4.64% (3.40-6.20%)									
2003	1,986	1,247	20.5	0.26	0.16	1.24% (0.94-1.66%)	7% (2-13%)	145 (48-243)	0.20% (0.06-0.36%)	38% (25-52%)	727 (484-1,017)	2.96% (1.74-4.66%)	55% (43-67%)	1,066 (824-1,308)	1.95% (1.27-2.94%)
2004	972	528	69.4	0.16	0.09	0.24% (0.17-0.35%)	17% (7-30%)	162 (65-292)	0.05% (0.02-0.10%)	60% (43-73%)	583 (421-713)	1.29% (0.72-2.22%)	23% (13-37%)	227 (130-356)	0.72% (0.35-1.39%)
2005	467	257	66.4	0.12	0.06	0.18% (0.13-0.23%)									
2006	572	518		0.40	0.36										
2007	188	176	39.4	0.10	0.09	0.25% (0.07-0.74%)									
2008	742	599	36.3	1.67	1.35	4.62% (1.83-11.54%)	7% (3-11%)	50 (25-83)	5.31% (1.04-45.0%)	53% (44-64%)	392 (325-467)	100% § (20.57-100%)	40% (31-49%)	292 (225-358)	1.98% (0.76-5.12%)
2009	254	178	9.6	0.29	0.21	3.08% (0.74-9.93%)	5% (2-58%)	12 (6-148)	4.17% (0.71-71.2%)	51% (37-63%)	130 (95-160)	6.92% (1.68-21.76%)	44% (33-58%)	112 (83-148)	1.82% (0.43-6.36%)
2010	1,118	639	42.7	1.88	1.07	4.40% (0.78-22.48%)									
2011	1,268	655	37.0	1.17	0.60	3.16% (0.42-20.96%)	16% (11-24%)	207 (138-300)	1.20% (0.15-8.25%)	51% (38-62%)	646 (484-784)	54.13% (3.81-100%)	33% (24-44%)	415 (300-553)	1.98% (0.24-15.33%)
2012	1,628	763	121.1	1.24	0.58	1.03% (0.34-3.00%)									
2013			81.6												
2014			12.8												
**Mean ± SD**	**2,987 ± 3,015**	**1,594 ± 1,570**	**117.0 ± 160.3**	**3.07 ± 8.33**	**1.69 ± 4.47**	**2.01 ± 1.75%**	**13.7 ± 7.3%**	**503 ± 665**	**1.59 ± 2.22%**	**51.2 ± 6.8%**	**1,363 ± 1,494**	**25.40 ± 37.84%**	**35.0 ± 11.8%**	**782 ± 687**	**1.74 ± 0.47%**
**Median**	**1,628**	**763**	**42.7**	**1.17**	**0.58**	**1.24%**	**16.3%**	**162**	**0.20%**	**52.8%**	**646**	**6.92%**	**32.7%**	**415**	**1.95%**

Table S6. Within-year differences in phenotype survival (*S*) based on random resampling (n=100,000 draws) of simulated phenotype survival estimates. The ratio represents the relative difference in survival rates from these draws and the p-value represents the fraction of draws where the difference in survival was greater than zero (n.s. = not significant)

Emigration year	Parr *S* : fry *S*		Parr *S* : smolt *S*		Smolt *S* : fry *S*	
	Ratio	p-value	Ratio	p-value	Ratio	p-value
1999	116.75	<0.0001	5.52	<0.0001	22.43	<0.0001
2000	21.30	<0.0001	1.09	n.s	20.82	<0.0001
2003	17.40	<0.0001	1.58	n.s	11.56	0.0010
2004	28.94	<0.0001	1.99	n.s	16.46	0.0021
2008	23.39	0.0096	50.32	<0.0001	0.59	n.s
2009	2.91	n.s	5.86	n.s	0.80	n.s
2011	86.40	0.011	52.49	0.024	5.02	n.s

Additional data table S1 (separate file; "Dataset 1.csv")

Reference strontium isotope (Sr^{87}/Sr^{86}) values, including otoliths from known-origin juvenile salmon (natal region of the otolith only) and water samples collected in the San Joaquin basin, freshwater Delta, and bays (Fig. 1). These data were used to estimate provenance and habitat transitions in adult salmon sampled on the Stanislaus River spawning grounds. Otolith data includes the mean, minimum, maximum, and standard deviation (SD) for all individual natal measurements for each fish.

References

Barnett-Johnson, R., C. Grimes, C. Royer, and C. Donohoe. 2007. Identifying the contribution of wild and hatchery Chinook salmon (*Oncorhynchus tshawytscha*) to the ocean fishery using otolith microstructure as natural tags. Canadian Journal of Fisheries and Aquatic Sciences **64**:1683-1692.

Barnett-Johnson, R., T. Pearson, F. Ramos, C. Grimes, and R. MacFarlane. 2008. Tracking natal origins of salmon using isotopes, otoliths, and landscape geology. Limnology and Oceanography **53**:1633-1642.

Cramer Fish Sciences. 2012. Juvenile Salmonid Out-migration Monitoring at Caswell Memorial State Park in the Lower Stanislaus River, California. 2010-2011 Biennial Report. Grant No. 813326G008.

Hobbs, J. A., L. S. Lewis, N. Ikemiyagi, T. Sommer, and R. D. Baxter. 2010. The use of otolith strontium isotopes ($^{87}Sr/^{86}Sr$) to identify nursery habitat for a threatened estuarine fish. Environmental Biology of Fishes **89**:557-569.

Kormos, B., M. Palmer-Zwahlen, and A. Low. 2012. Recovery of coded-wire tags from Chinook salmon in California's Central Valley Escapement and Ocean Harvest in 2010. Fisheries Branch Administrative Report 2012-02. Retrieved from: http://cahatcheryreview.com/bibliography/.

Manly, B. F. J. 2006. Randomization, bootstrap and Monte Carlo methods in biology. Chapman and Hall/CRC.

Mesick, C., D. Marston, and T. Heyne. 2009. Estimating recruitment for fall-run Chinook salmon populations in the Stanislaus, Tuolumne, and Merced Rivers. Instream Energy Flow Branch USFWS, http://www.waterboards.ca.gov/waterrights/water_issues/programs/bay_delta/deltaflow/cspa.shtml.

Pacific Fishery Management Council. 2016. Preseason Report I: Stock Abundance Analysis and Environmental Assessment Part 1 for 2016 Ocean Salmon Fishery Regulations. (Document prepared for the Council and its advisory entities.)

Palmer-Zwahlen, M., V. Gusman, and B. Kormos. 2018. Recovery of Coded-Wire Tags from Chinook Salmon in California's Central Valley Escapement, Inland Harvest, and Ocean Harvest in 2013.

Palmer-Zwahlen, M., and B. Kormos. 2013. Recovery of coded-wire tags from Chinook salmon in California's Central Valley Escapement and Ocean Harvest in 2011.

Palmer-Zwahlen, M., and B. Kormos. 2015. Recovery of Coded-Wire Tags from Chinook Salmon in California's Central Valley Escapement, Inland Harvest, and Ocean Harvest in 2012.

Peterson, M. L., A. N. Fuller, and D. Demko. 2017. Environmental factors associated with the upstream migration of fall-run Chinook salmon in a regulated river. North American Journal of Fisheries Management **37**:78-93.

Phillis, C. C., A. M. Sturrock, R. C. Johnson, and P. K. Weber. 2018. Endangered winter-run Chinook salmon rely on diverse rearing habitats in a highly altered landscape. Biological Conservation **217**:358-362.

Quinn, T. P. 1993. A review of homing and straying of wild and hatchery-produced salmon. Fisheries Research **18**:29-44.

Sturrock, A. M., J. D. Wikert, T. Heyne, C. Mesick, A. E. Hubbard, T. M. Hinkelman, P. K. Weber, G. E. Whitman, J. J. Glessner, and R. C. Johnson. 2015. Reconstructing the Migratory Behavior and Long-Term Survivorship of Juvenile Chinook Salmon under Contrasting Hydrologic Regimes. PLoS One **10**:e0122380.

Sturrock, A. M., Satterthwaite, W. H., Cervantes-Yoshida, K. M., Huber, E. R., Sturrock, H. J. W., Nusslé, S., & Carlson, S. M. (2019). Eight Decades of Hatchery Salmon Releases in the California Central Valley: Factors Influencing Straying and Resilience. Fisheries, 44(9), 433-444. doi:10.1002/fsh.10267

USFWS. 2017. "Chinookprod" database. Unpublished database maintained by the Anadromous Fish Restoration Program and Comprehensive Assessment and Monitoring Program, Lodi and Sacramento, California.
